# A contemporary class structure: Capital disparities in The Netherlands

**DOI:** 10.1371/journal.pone.0296443

**Published:** 2024-01-31

**Authors:** J. Cok Vrooman, Jeroen Boelhouwer, Mérove Gijsberts

**Affiliations:** 1 The Netherlands Institute for Social Research|SCP, The Hague, The Netherlands; 2 Department of Sociology, Utrecht University, Utrecht, The Netherlands; 3 Department of Interdisciplinary Social Science, Utrecht University, Utrecht, The Netherlands; University Putra Malaysia: Universiti Putra Malaysia, MALAYSIA

## Abstract

The academic and public debate on social inequality has recently been fuelled by large disparities in income and wealth, profound changes in the labour market, and other emerging cleavages in post-industrial societies. This article contributes to the discussion by arguing that class divisions are theoretically based on four types of capital: people’s economic means, their social capital, their cultural resources, and the combination of their health and attractiveness (‘person capital’). From this premise, the social structure of the Netherlands is examined. A dedicated survey was linked to microdata from the national population register, tax authorities and benefit agencies. Using latent class analysis, we assess contingencies in the distribution of the different resources, and identify a structure consisting of six capital groups. The established upper echelon (15.5% of the adult population) has the most capital, followed by the privileged younger people (12.7%), the employed middle echelon (26.9%) and the comfortable retirees (16.6%). Total capital is lowest among the insecure workers (13.5%) and the precariat (14.8%). Each social class has a distinctive mix of the four types of capital, highlighting the need to look beyond economic differences in order to comprehend structural inequality. The results of this study also indicate that resource disparities between classes coincide with other forms of social hierarchy and contrasts by age. Moreover, the contemporary class structure is associated with divergent views and experiences among the Dutch. Classes with little capital tend to rate politics, society, and their own social position more negatively. In addition, they value self-enhancement and hedonism less than today’s upper classes and report lower levels of well-being.

## 1. Introduction

While economists often consider social disparities in terms of poverty, the distribution of income and wealth, and intergenerational income mobility [1–4], sociologists tend to attribute a pivotal role to the occupational structure [5: 15–25, 67–71]. In the economic perspective the emphasis on financial inequality is related to the discipline’s traditional focus on the maximisation of material welfare and the fulfillment of individual needs or preferences [6, 7], the role of incentives and rent-seeking in the rational behaviour of economic actors [8–10], the institutionalisation of property rights in capitalist society [11], and the importance of material consumption as a signal of economic success and power [12, 13]. In sociology, on the other hand, neo-Weberian theory assumed the existence of a social hierarchy, based on ‘a set of principles […which] allocates positions to classes so as to capture the major dimensions of differentiation in labour markets and production units that are consequential for the distribution of life chances’ [14: 39]. This approach resulted in the identification of distinct occupational ‘Big Classes’–skilled and unskilled workers, the petty bourgeoisie, the service class etc.–associated with the late industrial era [15]; and, alternatively, in ‘gradualism’, in which a large number of occupations are ranked according to their prestige and socio-economic status [16–19].

For various reasons, both disciplines have witnessed a revival of the debate on inequality and social structure in recent years. In many advanced societies, income differentials have widened over the past few decades. These have been exacerbated by the global economic recession that began in 2007; and while pandemics tend to reduce income inequality, the effects of the COVID-19 period may be atypical [20–22]. Wealth inequality in general has increased dramatically since the 1980s, partly due to rising house prices and class-specific within-family transfers [23–25]. The growing number of people with extremely high incomes and fortunes is unlikely to reflect exceptional talent or achievement among the business elite. Economic deregulation, market concentration and new information technologies probably evoked ‘winner takes all’-mechanisms–very high marginal returns at the top–that were amplified by rent-seeking and cronyism. Under these conditions, the acquisition of exceptionally large sums of money may have come to depend not on merit, but on luck, self-serving behaviour, and access to influential persons and organisations [26–28].

In the labour markets of most advanced economies, the share of total employment accounted for by industrial production, agriculture, fishing and mining has declined over time. The service sector has grown, with a large increase in the number of well-paid managers, financial and technical specialists, and urban creatives. These higher professionals often work in high-tech companies, banks, media, advertising, universities etc. [29, 30]. At the bottom of the labour market more people became employed in low-skilled jobs in fast food, hospitality, cleaning, transport, care etc. Such jobs partly cater to the needs of the new professionals (e.g. parcel and meal deliveries, tourism, childcare, hairdressing, dry cleaning, Uber taxis). They tend to be poorly paid, and offer little in terms of job security and other working conditions: psychological stress is high, autonomy is limited, and there are few opportunities for skills development, personal growth, and upward mobility. Some authors therefore equate their growth with the emergence of a post-industrial service proletariat or new ‘precariat’, in which migrant workers, youth and women are over-represented [31–34].

Moreover, in many societies, a significant proportion of the adult population is out of work nowadays, and dependent on various social security schemes that only partionally reflect their former occupational status [35, 36]. According to De Swaan the modern welfare state introduced new categories into the class structure, such as pensioners, welfare clients and other benefit recipients [37]. Since the 1980s, these groups have typically experienced a decline in social protection due to austerity measures and the marketisation of social security [38–40]. The inequality debate has also been fuelled by demographic transitions: migration processes, population ageing and declining fertility. Traditional breadwinner and three-generation households became less common, while the share of single persons, one-parent families and dual-earner households increased [41]. Finally, recent technological innovations–social media, internet-based platforms, artificial intelligence, robotics–may have changed labour demand and the occupational structure [42–45]. Emerging technologies can also alter the distribution of life chances by redefining how people should think, look and behave [46, 47], and by providing companies and governments with extensive opportunities to monitor workers, consumers and citizens [48, 49].

This contribution explores whether these developments have crystallised into a new post-industrial class structure. After an overview of potentially emergent forms of inequality, several empirical analyses are presented for the Netherlands, which is an interesting test case for several reasons (see section 3.3). First, we assess whether the Dutch population is divided into social classes based on individual resource disparities. We then consider whether such a class structure is connected with other forms of social distinction (such as a person’s age, occupational class, gender and ethnic background) and with differences in well-being, personal values and conceptions of society.

## 2. New forms of inequality

The developments discussed in the previous section may have affected the contemporary social hierarchy; and indeed the literature suggests various new forms of segmentation. Some of these relate to changes in the classic dimensions of inequality in economics and sociology: new disparities in wealth, income and the occupational structure. Other authors, however, argue that certain new dimensions have become crucial to the contemporary allocation of social positions. These concern cognitive stratification; individual life styles and identities; attractiveness; health inequalities; and multidimensional capital disparities.

### 2.1. Emerging disparities in wealth, income and the occupational structure

Some economists emphasise that since the 1980s financial assets have become increasingly important for the distribution of life chances. In their view, a path towards a polarised class structure based on wealth is materializing, with a sharp distinction between a small group of very rich people at the top and the rest of the population. Piketty regards the higher growth rate of financial capital (relative to economic output and wages) as the main factor driving this process, combined with a decline in fiscal redistribution and the opportunity to transfer private fortunes largely intact across generations [50]. Stiglitz attributes it to a lack of regulation of market imperfections: information asymmetry, the formation of cartels and oligopolies, and price fixing. In his view, this is driven by a preference among mainstream economists and policy makers for non-intervention by the state; a concentration of economic power in the hands of the wealthiest top-1%; and the interconnectedness of the economic and political elites, as evidenced by lobbying, party funding, and job-hopping between politics and the private sector [51, 52].

Others, largely for the same reasons, refer to growing income differentials in recent decades, with a burgeoning group of very high earners, middle incomes falling behind and the number of ‘working poor’ on the rise [53–55]. Mian *et al*. note that in the USA, more income inequality translated into higher savings at the top and lower interest rates. According to these authors, this implies that growing income and wealth disparities have become mutually reinforcing to a greater extent than before [56]. Wilkinson and Pickett suggest that high income inequality is at the root of a plethora of social issues [57]: health problems and low life expectancy, drug abuse, teenage pregnancy, low social cohesion, violence and imprisonment. They attribute this to the increased status competition and the ensuing social anxiety resulting from large income differentials, and some empirical support for this mechanism has been found [58].

With regard to the labour market, recent economic literature suggests a trend towards job polarisation based on routine-biased technological change [59–63]. In this line of reasoning, jobs in the middle segment often consist of repetitive and standardised tasks (e.g. accounting), and their routine nature makes them susceptible to replacement by digital technology. Higher occupations, on the other hand, are typically non-routine and cognitive; and here ‘computerisation’ tends to increase productivity and rewards, leading to higher incomes. In non-routine jobs at the bottom of the labour market, such as cleaning, this type of technology presumably plays a limited role. The resulting occupational polarisation, reinforced by the trend towards offshore production [64], is seen as the main driver of growing income inequality. Fernández-Macías and Hurley, however, found that job polarisation is not a universal trend across European countries, attributing this to the complex interaction of technological change with diverging institutional and cultural contexts [65].

Sørensen emphasised the impact of institutional change on labour market positions and the distribution of earned incomes [66, 67]. He postulated that the decline in trade union membership and collective bargaining in liberalised economies [68] made wages more dependent on individual productivity and personal endowments. This would lead to greater inequality of earnings within occupational groups, and thereby erode traditional divisions between Big Classes; yet Williams found no evidence of this trend in the British case [69].

Grusky and his colleagues proposed an alternative approach of social stratification–in addition to Big Classes and gradualism–through ‘micro-classes’ [70–72]. These typically consist of more than 100 specific job categories (e.g. medical doctors, lawyers, actors, artists, politicians, carpenters, mechanics, truck drivers). Micro-classes are likely to be socially closed from one generation to the next because parents provide their offspring with job-related resources: occupation-specific skills (such as acting or carpentry), cultures and tastes (the aspiration to become a medical doctor), and networks that have been developed through parental interactions in the workplace. They may also pass on occupational assets to their children, such as the family business or farm. Processes of ‘occupational reproduction’ could therefore be the main mechanism underlying low intergenerational mobility [73]. However, occupational micro-classes predict people’s lifestyles and socio-political attitudes better than their educational attainment, income and wealth [71]. In addition, Erikson *et al*. found that ‘inheritance’ through micro-classes explained less intergenerational mobility than did Big Class distinctions, and was less common among women than among men [74].

### 2.2. From meritocratic narrative to cognitive stratification

Modernisation theory presumed that, in complex industrial societies, the allocation of social positions through ascribed characteristics (parental status, kinship ties, gender, ethnic background) was no longer efficient [75–77]. It therefore had to give way to selection based on personal qualities (intelligence, talent, effort, motivation) and achievements (educational attainment, skills, knowledge and experience). The shift towards such a meritocratic allocation process was facilitated by the expansion of the education system, the rise of objective assessment procedures–elaborate testing of pupils and students, human resource management–and, supposedly, by a decline in labour market discrimination, patronage and nepotism. As a result, intergenerational mobility should increase over time, and the direct link between people’s social origin and their educational and occupational position would necessarily become weaker [78]. Any remaining inequalities in educational attainment, occupational status, income, wealth and other social outcomes would ultimately reflect differences in talents and achievements, and could therefore be considered fair and legitimate [79].

While popular belief in meritocracy remains widespread, particularly in unequal societies [80], this meritocratic narrative has been challenged in the literature. Empirically, the ascription of social positions by ethnic background and gender has not been eradicated, especially in the labour market [81, 82]. Absolute intergenerational mobility has tended to evolve as expected: over time, children have generally attained higher levels of education and employment than their ancestors [83, 84]. However, the relative position of parents and children within their own cohorts–taking into account educational expansion and changes in labour supply and demand–appears to have remained fairly stable. In this sense, society has not become more open [85–87]. Over the life course, the occupational level of the father has become less relevant for children’s educational attainment and occupational careers, as predicted by modernisation theory. The overall effect of parental education, on the other hand, has changed little. There has been a general decline in its impact on their offspring’s primary school achievement, but an increase in its effects on secondary and tertiary education [88].

Sandel argues that educational grades nowadays determine labour market opportunities and life chances [89], and Murray points to their impact on people’s wealth and the values to which they subscribe, such as work ethic and honesty [90]. From their perspective, ‘cognitive stratification’–a social hierarchy based on cognitive ability and educational attainment–has over time become an inevitable consequence of meritocratic selection and educational expansion, as predicted by Young [91]. Cognitive stratification could be driven by various factors. These include the selection of a cognitive elite by the education system through high school fees, entrance tests, honours programmes and elite colleges; the pooling of resources and genetic (dis)advantages through educational homogamy; and the differential investment of highly and low-educated parents in the school career and cultural capital of their offspring, e.g. through private tutoring, studying abroad and distinctive leisure activities [92–96].

### 2.3. Liquidity and the decline of social class: individual life styles and identities

Postmodernist theory claims that social classes have become less recognisable in recent decades. This is thought to result from a condition of reflexive or liquid modernity in contemporary societies [97–102]. As economic, social and technological systems became interconnected on a global scale, new risks emerged that were difficult to predict or control: nuclear threats, climate change, pandemics, the outsourcing of economic production, the growth of transnational corporate power, etc. This created anxieties and reflexive doubts about the ability of states, institutions and experts to manage such risks, even in societies that by traditional standards offer a high degree of social protection [103, 104]. Under these circumstances people’s lives also became more liquid. On the labour market they experienced an increase in flexible, temporary and low-quality employment and greater income volatility. Social relations became more fragmented and unstable, as the classic heterosexual breadwinner family gave way to a variety of other household types that could fluctuate over the life course: being single, one-parent and combined families, co-residents, and same-sex cohabition and marriage. In conjunction with increasing spatial mobility, this brought about a decline in social cohesion at the community level. It has therefore become more important to construct individual life styles and identities, particularly through consumptive behaviour and, more recently, social media presence. In this line of reasoning, life chances no longer depend on one’s origins or merits, but on personal choices in liquid conditions. It is postulated that, as a result, traditional distinctions of social class–both in terms of structural positions and in the ideas that people hold–are gradually dissolving, and individual dynamics now prevail. Bauman takes the most radical position on the liquefaction of modern society [105]. In his view, social status has come to depend on consumption patterns, with the main social distinction being between those with affluent and cosmopolitan life styles, and those who aspire to the same but cannot afford it. Bauman’s approach, however, has been criticised for its wide-ranging theoretical assumptions and limited empirical substantiation. It neglects the possibility that traditional social structures may persist at a deeper level [106–108]. In addition, processes of ‘re-embedding’ may occur, where new distinctions emerge reflecting differences in socio-economic position, ethnic background and power relations under liquid societal conditions [109: 656–659].

### 2.4. Attractiveness and aestheticisation

Rubenstein noted that “in twentieth-century American society, physical beauty emerged as a resource, like wealth or talent” [110: 212]. A meta-analysis of more than 900 psychological studies found that attractiveness is related to children’s and adults’ treatment (e.g., attention and help received), behaviour (e.g. social skills, adjustment), and various social outcomes (e.g., popularity, health, sexual partners, occupational success) [111]. This is supported by the substantial economic literature demonstrating the existence of a ‘beauty premium’ in the labour market, and career and earnings penalties for plain-looking and unattractive persons [112–120]. This line of research tends to consider an individual’s height, weight and facial symmetry as the key beauty traits that influence their occupational and financial position.

Kanazawa and Still suggest three underlying mechanisms [121]: discrimination on the basis of appearance by employers, co-workers, or customers; self-selection of attractive people, who choose labour market sectors where they can marketise their beauty; and the use of individual variety in attractiveness as a proxy indicator of someone’s health and productivity. In sociology, Simmel already pointed out that different styles of dress reflect diverging class positions and serve the dual purpose of conforming to social standards and emphasising one’s individuality [122]. The more recent sociological literature views attractiveness as a status cue that affects patterns of interaction [123], and has introduced the notion of aesthetic capital: “traits of beauty that are perceived as assets capable of yielding privilege, opportunity and wealth” [124: 566]. The sociological approach points not only to labour market and income differences related to beauty. On top of that, aesthetic capital is relevant to friendship relations, partner selection and the willingness of others to offer help [125–133]. Moreover, here the demarcation of attractiveness goes beyond the face and body shape. It also refers to other physical characteristics (skin colour, muscularity, smell, tone of voice, the condition of one’s teeth, wrinkles, scars, disfigurement), various aspects of grooming (such as clothing, shoes, hairstyle, make-up, jewellery, piercings, tattoos), erotic appeal and psychological traits like charm, likeability and salesmanship [134–139].

Several authors have argued that a process of ‘aestheticisation’ is taking place in contemporary societies [140–142]. Following the postmodernist line of reasoning discussed above, this could be due to the liquefaction of traditional class criteria, making people’s appearance more important in signalling their identity, social position and aspirations. From an economic point of view, aestheticisation could be driven by the dominant consumer culture [143] and by the growing size and influence–partly through advertising–of aesthetic producers in capitalist societies: the multinational fashion and beauty industries, elective cosmetic surgery clinics, fitness schools, spas, massage parlours, hair and nail salons, diet, wellness and mindfulness course providers, etc. [144–146]. Furthermore, many professions now require ‘aesthetic labour’ [142, 147]. Public display of the ‘right’ looks, attitudes and behaviours is essential for employment in hospitality and retail, finance and law, television and social media, and politics [148–151]. Technological developments (mobile phones and other forms of digital connectivity) have made it important to be ‘camera-ready’ at all times, and may have contributed to a global standardisation of certain normative body types. Widdows suggests that a young, firm, smooth and thin physique has become an almost universal moral ideal [152]. A counter-movement challenging the objectification of the (female) physique and advocating ‘body positivity’ does not seem to have had much impact yet [153–155]. In aestheticised societies, attractiveness and ugliness are likely to be constitutive elements of the class structure.

### 2.5. Health and inequality

Over time and across nations, there is a consistent and well-documented link between socio-economic status and health: people with less education, lower jobs and limited income generally live shorter and have a higher prevalence of disease and disability [156, 157]. In the literature, this is often attributed to social causation [158–162]. Those at the bottom of society have less favourable circumstances: they are more exposed to environmental health risks (substandard housing, air pollution, hazardous working conditions), have less access to adequate health care, are less able to maintain a healthy life style and experience more chronic stress [163]. As a result, they end up with more medical problems than those in higher positions, and a socio-economic health gradient emerges. Others, however, argue that health differences generate social inequalities: people’s physical and mental condition can have a major impact on their life chances [164–168]. According to this selection hypothesis, individuals with serious medical issues will generally find it more difficult than healthy persons to achieve higher levels of education, to have successful careers, and to become high earners. This is because disabled men and women tend to need care and facilities that may not be available, and their impediments do not always allow them to study or work full-time. In addition, organisations can be less inclined to hire or promote unhealthy persons, and more likely to dismiss them. Employers may assume that they are less productive and more prone to sickness absenteeism than healthy workers; that they require a lot of counselling; that they increase the administrative burden; or that they do not fit into the work process or corporate culture [169–172]. From this perspective, people’s physical and mental state is a key determinant of their social position.

Empirical evidence on the mechanisms of social causation and health selection is mixed, but recent systematic reviews suggest that both processes play a role. Which one dominates depends on the life stages and aspects of socio-economic status being studied [173, 174]. Theoretically this calls for a reconceptualisation in which health is recognised as a distinct element in the generation of social disparities [157, 175]. This can build on the earlier work in health economics, where a person’s mental and physical condition is seen as a resource [176–179] linked to other forms of capital [180–183]. Health should then preferably be analysed from a life course perspective that assumes a “dynamic interplay between different social determinants and health statuses, where the relationship can be ‘causal’ during one phase and ‘selective’ during the next” [184: 619].

### 2.6. Multidimensional capital disparities

Recent sociological research suggests that a complex layered social structure has evolved, based on different types of resources [185–187]. It argues that analysing hierarchy in complex societies requires “a concept of class which does not reduce it to a technical measure of a single variable and which recognises how multiple axes of inequality can crystallise as social classes” [188: 1011]. Inspired by the work of Bourdieu [189–191], these studies start from the assumption that social classes are not merely economic phenomena, but are also subtly related to selection processes that operate through cultural distinctions and social networks. In theory, economic disparities need not coincide with cultural and social resources: differences in occupational status, income or wealth may even be inversely related to certain group-specific cultural practices and network characteristics [191: 21]. A multidimensional approach could therefore identify a finer-grained and more meaningful structure than schemes based purely on people’s income, wealth or occupational status. Classes would then be characterised by divergent combinations of economic, cultural and social capital stocks.

From a latent profile analysis of indicators of these three types of resources, Savage *et al*. conclude that there are currently seven social classes in the United Kingdom [185]: the elite (6% of the population), the established middle class (25%), the technical middle class (6%), the new affluent workers (15%), the traditional working class (14%), the emergent service workers (19%) and the precariat (15%). Sheppard and Biddle used the same methodology for Australia, which is often regarded as a more egalitarian society than the UK, with a more comprehensive ‘Antipodean’ welfare state [187, 192, 193]. Nevertheless, they found a similar class structure, albeit with slightly different group proportions, and the new affluent workers and the emergent service workers ending up in a single class. For Croatia, which offers a quite different historical and institutional context, Doolan & Tonković examined the distribution of economic, social and cultural capital through multiple correspondence analysis and identified six resource classes. The least resourceful group consists of older people (mostly women) with primary education and inadequate state pensions [194].

## 3. Materials and methods

### 3.1. Conceptualisation of four types of capital

Following Savage *et al*. and Friedman and Laurison [188, 195], we assume that current forms of social inequality reflect not only differences in economic resources, but also in people’s compatibility with certain contexts (cultural capital), and in the help they can obtain from others (social capital). To this we add person capital, which refers to an individual’s health and attractiveness. By including ‘how people fit in’, ‘who they know’ and ‘who they are’ in the theoretical framework, we aim to measure multidimensional social inequality in line with the debates outlined in the previous section.

### Economic capital

The first type of resource we distinguish is economic capital. This theoretically consists of one’s educational attainment and professional skills, labour market position, and income and wealth. The distribution of these resources may reflect traditional forms of economic inequality (disparities in labour market positions and income, meritocratic allocation), but also more recent manifestations, such as growing wealth differences and cognitive stratification. It should be noted that we regard educational attainment primarily as an economic resource, because it signifies the knowledge, skills and labour market qualifications people have acquired. This is in line with Becker’s human capital theory [196]. Educational inequalities may subsequently translate into different levels of income, wealth, health, social relations and cultural capital. In our approach these resources should be measured directly, and their contingency with the level of education is an empirical matter. We therefore do not share Bourdieu’s assumption that educational attainment is by definition a form of ‘institutionalised cultural capital’. Formal education can theoretically reproduce the existing social order by installing class-specific forms of cultural capital in its students. But this is not inevitable: modern education systems often have explicit tasks relating to talent development and the provision of equal opportunities for all [197], and these can generate upward social mobility.

### Cultural capital

A second type of resource is cultural capital, i.e. collective predispositions, expressive behaviours and attributes that mark social positions. It can take three forms: language and communication (a person’s accent, dialect or vocabulary; the ability to speak foreign languages; digital literacy); tastes, preferences and cultural knowledge (e.g. attending and appreciating classical concerts or theatre productions); and symbolic attributes (reputation and celebrity, formal titles and honours). This largely builds on the work of Bourdieu. Theoretically, however, this type of resource is not confined to the predispositions, behaviours and attributes of ‘high’ culture, but also includes emerging forms, such as preferences for alternative music styles, cultural omnivorism, and ecologically responsible life styles [198–204]. That tallies with Dressler’s notion of cultural consonance, which refers to people’s ability to live up to the valued aspects of a certain domain in their society [205]. Cultural capital can thus be context-specific: it may depend on the circles in which individuals move and the circumstances in which they find themselves. In particular, the possession of the right kind of cultural capital can be crucial for attaining higher social positions. Elites and the upper-middle classes may achieve social closure by using it as a screening device [206–208]. Newcomers, on the other hand, can eliminate themselves because their lack of cultural capital may lead them to stay out of the higher circles of society, to question the social mores prevailing there, or to avoid ‘risky’ career choices [195].

### Social capital

Social capital consists of the resources that are embedded in the relationships individuals have with others. It refers to one’s position in social networks, and the size and quality of those networks. Network assets can relate to financial and material support: contacts who are able and willing to provide money, time, goods and services. They can also consist of the provision of information (e.g. about suitable marriage candidates or job openings), influence (e.g. a connection who has a say in hiring decisions), or social credentials (someone who can vouch for you). Further resources others can provide are emotional support–trusted friends and family who offer their sympathy, understanding and solidarity–and the recognition of a person’s identity and group membership [209, 210]. We take an ego-centred approach here: social capital is something that an individual can possess, rather than a characteristic of neighbourhoods, religious groups, the civil society, regions, or entire nations [211]. An extensive sociological literature indicates that this type of resource plays an important role in the social hierarchy [190, 212–221]. People at the top and at the bottom of society tend to differ in the size and quality of their networks. Those at the top may be well-connected from the outset because of the acquaintances that children gain from their families. Building on these and other resources, this may translate in later life into a greater ability to invest in their social relationships, to access and mobilise social capital when needed, and to generate returns on their contacts with others (e.g. in terms of educational attainment, occupational careers, income and well-being). Homophily and opportunity hoarding may also be responsible for structural differences in resources. People tend to associate with others who have similar backgrounds and life styles, and powerful networks may seek to monopolise resources and deny access to others [222–224].

### Person capital

Person capital comprises the (dis)advantages of an individual’s bodily and mental state, and this concept may allow us to capture some of the more recent distinctions of post-industrial societies. Our conceptualisation builds in part on the work of Bourdieu, who saw ‘embodied’ capital as a specific cultural resource. He referred to physical or mental dispositions that people acquire and develop in response to their social background [190]. In health economics, this is sometimes referred to as psychophysical capital [225]. However, our notion of person capital also harks back to Pareto’s somewhat neglected proposition that life chances depend on social competition based on individual heterogeneity [226], and not exclusively on group characteristics: ‘Whether certain theorists like it or not, the fact is that human society is not a homogeneous thing, that individuals are physically, morally, and intellectually different’ [227: 1419]. This leads us to consider person capital as an independent theoretical dimension of social inequality: two young individuals with similar economic, cultural and social resources may end up quite differently in life if one is chronically ill or unattractive and the other is not. We divide it into three subtypes: physical capital (bodily health and abilities), mental capital (psychological health and abilities) and aesthetic capital (individual traits that are attractive to others, such as beauty; the right attitudes and behaviours). The distribution of such characteristics may reflect people’s past and present circumstances, but can also be rooted in genetic differences between individuals [228–230]. Person capital may be generic (a healthy person is usually in an advantageous position) or context-specific (e.g. different dress codes apply in nightlife and during a job interview for a managerial position). The inclusion of person capital as a separate dimension reflects the growing literature on health inequalities, attractiveness and the aestheticisation of society. It may also be informative in relation to postmodernist theory on the individualisation of life styles, e.g. through personal branding and digital identities. If the liquefaction hypothesis discussed earlier is correct, hyper-individualisation should occur and we would not expect to find a clear class structure.

### Multidimensional correspondence of capital

Our starting point is that social classes can be thought of as groups with distinct mixes of the four types of capital. These theoretically determine the life chances of their members, are typically linked to the historical division of power and interests, and may be associated with different worldviews (ideologies, values, social norms, policy preferences). The multiple correspondence of the four types of resources implies that two classes can have similar stocks of total capital, but still be meaningfully different due to its composition. This would be the case, for example, if both have abundant social and cultural capital, but one social class has far more income and wealth, while the other is much healthier and more attractive. Whether or not ‘classical’ resource dimensions (income, wealth, education, occupation) dominate the multidimensional structure is an empirical issue. This may vary with the societal context (e.g. different welfare regimes and policy trends, the cycle of economic booms and busts). Rather than assuming a stable predetermination of social positions based on one’s initial educational achievements and subsequent place in the occupational hierarchy–with other resources mostly embedded in this socio-economic status–we regard the (sub)varieties of capital as dynamically interrelated. Resources can therefore (dis)accumulate over time–but not necessarily in a linear fashion, or in the same way for each type of capital or social class. In addition, the relative impact of the four types of capital on an individual’s opportunities can vary over the life course. It is conceivable that educational achievements, knowing the right persons and attractiveness are decisive for starting a career or finding a partner among younger adults, whereas physical health and the size and quality of networks offering informal care can become crucial as people grow old. This implies that individuals not necessarily belong to the same resource class throughout their lives. Finally, the notion of ‘capital’ implies that different types of resources are convertible into each other. This would for instance occur if affluent classes consistently spend more money on cultural activities, maintaining social networks, and the well-being and appearance of themselves and their children. The resulting advantage in non-economic resources may subsequently translate into higher earnings and wealth (or less financial depletion in bad times), thus perpetuating the economic privileges of these social classes [190, 231–236].

### 3.2. Research questions

Using data from the ‘Disparities in the Netherlands’ project, we attempt to assess the nature of the contemporary class structure and its associated characteristics in an affluent Western society. Our first research question is *whether*, *on the basis of group differences in the four types of capital*, *contemporary Dutch society is divided into social classes*.We consider class structuration to be more pervasive when the number of classes is small (low fragmentation); when their members consistently possess divergent and interpretable combinations of different types of capital (high multiple correspondence); and when the resource disparities between higher and lower classes are large (wide scope) and do not change over time (stability). A division into two groups, whose members are resourceful or not across the board, and where the resource differences between the upper and lower classes remain stable over the years and from one generation to the next, is therefore sharper than a volatile configuration of six groups with partly overlapping resources and limited capital differences between the two extremes.

If there are social classes based on resource disparities, they may be related to other forms of inequality. In general, we should a priori expect traditionally vulnerable groups to have fewer resources and the privileged to have more. Social divisions in terms of age, labour market characteristics, gender, ethnic background, household composition and religion may then turn out to be (partly) class issues. Drawing on the traditional materialist argument that objective social positions are an important determinant of political behaviour [237], the class structure and related forms of inequality might also translate into diverging voting patterns. On the other hand, it is conceivable that the multidimensional nature of our resource approach and the intersectionality of other forms of inequality [238, 239] will lead to rather complicated empirical links and subtle conclusions. Our second research question is therefore open and exploratory: *To what extent do resource-based class differences coincide with other social distinctions (age*, *labour relations*, *ethnic background*, *gender*, *religion*, *household composition*, *voting intention)*?

The social classes of the late industrial era were closely linked to specific world views. These were partly socialised through group-based organisations (trade unions, political parties, religious pillars), which ensured, for example, that the working class was also a cultural community. According to Grusky and Hill such ‘ideological work’ has largely disappeared, as its traditional agents have become less powerful, and shifted their attention from class-based action to issue politics and the provision of tangible benefits to their members [79: 2]. This would lead us to expect that the relationship between class positions and people’s subjective experiences has become rather weak today. On the other hand, several recent developments may have counteracted this. The decline of economic security and industrial employment sometimes disrupted local communities (e.g. in the American ‘Rust Belt’), which possibly shows in varying degrees of societal pessimism and political discontent [90, 240–242]. In addition, the growth of social media has opened up new channels of communication and socialisation, and has been instrumental, for example, in the polarisation and culture wars between Democratic and Republican elites and partisan voters in the USA, and between Brexiteers and Remainers in the UK [243–249]. Similar issues are also raised in the Dutch public debate [250–253]. Through such recent trajectories class distinctions might still coincide with divergent perceptions. It is therefore worth exploring whether the contemporary class structure is linked to subjective ideas and experiences: how people view society and politics; their sense of belonging; their trust in others; their satisfaction with life and whether they feel able to make ends meet; and what they value and pursue. This leads to our third research question: *To what extent do resource-based class differences correspond to divergent socio-political views*, *subjective well-being and personal values*?

### 3.3. The Netherlands as a test case

The Netherlands is an interesting test case for investigating new forms of inequality for a number of reasons (see also S1 Text). Many of the drivers theoretically associated with postmodern social disparities are present. Over time, the service sector has grown and new labour market distinctions have emerged, partly as a result of the increasing number of people without full-time permanent contracts. The population shares of pensioners and benefit recipients are considerable. The country has a globalised and digitally advanced coordinated market economy that combines prosperity with large wealth inequalities and growing in-work poverty. Various demographic changes have occurred and are continuing (ageing and migration processes), and the degree of de-traditionalisation (educational expansion, secularisation, gender equality, progressive ethics) is high. In addition, the extensive institutional regime and the fragmented, yet ultimately collaborative, political system provide a different context than in Anglo-Saxon countries, which may also affect the social hierarchy: the contemporary class structure in the Netherlands, or the size of certain classes, may not be the same as in the United Kingdom, Australia or the USA.

### 3.4. Data

The survey ‘Disparities in the Netherlands’ (known by its Dutch acronym ViN’14) was conducted in 2014 among 2,952 Dutch citizens aged 18 and over. It was based on a new stratified random sample that was drawn by Statistics Netherlands (CBS) from the national population register. To ensure adequate representation of high and low income groups, members of the bottom and top deciles of the standardised disposable household income distribution (and within the latter, individuals from the top 1%) were oversampled. Potential respondents were personally invited by letter to participate in a survey on what Dutch society looks like (e.g. differences between young and old, healthy and unhealthy persons, and people with high and low levels of education). The online and written versions of the questionnaire were pre-tested for clarity and consistency through ten cognitive interviews with a test group that was heterogeneous in terms of gender, age, education level and income. A mixed mode data collection process was used, with 56% of respondents completing the questionnaire online (using a personal code) and the remainder opting for the written version. The fieldwork was conducted by I&O Research and, after using various incentives (gift vouchers, a prize draw for a number of iPads) and numerous written and telephone reminders, achieved a response rate of 43% [254]. Some population groups were by definition not represented in the survey, such as homeless persons without a postal address, and we do not have information on the participation of individuals with low literacy or who do not speak Dutch. Compared with the original sample, migrants and young people are slightly under-represented in the response group (a deviation of -0.2 to -0.6 percentage points). ViN’14 was weighted by CBS in terms of gender, age, ethnic origin, family composition, level of education and degree of urbanisation. The weights also correct for the oversampling of respondents at the top and bottom of the income distribution. *Post hoc* the survey data were anonymously linked to CBS microdata (2011–2014) covering all Dutch citizens, based on the population register and information from the tax and benefit authorities. This made it possible to add variables on income, wealth, age, gender, household type and ethnic origin. Data collection was carried out in accordance with the legal and ethical codes to which Statistics Netherlands, SCP and I&O Research are bound.

ViN’14 includes indicators for most aspects of the four variants of capital that are the subject of the first research question (see Table 1; detailed descriptions of the survey items are provided in S2 Text). For economic capital, these are educational attainment, current labour market position, disposable household income, liquid household assets and home equity. Regarding cultural capital, tastes and preferences are captured by a life style scale in the Bourdieusian tradition: did the respondent go on a holiday abroad in the past year, dined in a restaurant costing more than 100 euros per person, and visited forms of ‘high’ culture such as classical concerts, theatres, art galleries and museums? There are two indicators for ‘language and communication’. We consider proficiency in English as an indicator of the Dutch people’s familiarity with the hypercentral language of global business, science and culture [255, 256]. A self-assessment of English proficiency is available in the survey. This was based on the classification of the Common European Framework of Reference for Languages and condensed into five categories. Digital literacy is a nascent form of cultural resources; in his later work, Bourdieu saw it as an element of ‘technical capital’, a specific sub-type [257–259]. These skills were determined at a basic level using a scale derived from three questionnaire items: are respondents able to use a word processor, to install a computer program and set up security on their PC? ViN’14 does not contain information on the symbolic aspects of cultural capital (reputation, titles and awards).

**Table 1:** Measurement of four types of capital.

Type and elements of capital	Categories
Economic capital	
educational attainment	primary; lower secondary; higher secondary; tertiary
current labour market position	inactive; unemployed; (pre)retired; student; self-employment; paid employment
disposable household income*	decile 1; 2–3; 4–5; 6–9; percentile 90–99; percentile 100
liquid household assets*	negative; €0-5k; €5-50k; €50-500k; >€500k
home equity*	negative; renter; positive first tercile; second tercile; third tercile
Cultural capital	
life style	scale: holidays abroad; expensive restaurants; visits to ’higher’ culture (terciles)
basic digital skills	scale: ability to install software; to set up PC security; to use a word processor (0–3)
mastery of English language	none; limited; fair; good; mother tongue
Social capital	
strong ties	scale: frequency of contact with family, friends and neighbours (in 5 categories)
size of core discussion network	number of people to discuss personal matters with (in 5 categories)
access to resourceful positions	scale: number of people with influential occupations one knows (0–4 or more)
Person capital	
physical capital	scale: subjective health, difficulty climbing stairs (terciles)
mental capital	scale: self-confidence; negative self-image; suffering from depression (terciles)
aesthetic capital	scale: self-rated appearance; perceived rating of appearance by others (terciles)
body mass index	severely overweight; overweight; underweight; healthy weight

* Based on administrative data linked to the survey. Disposable income has been standardised for different household types using the Statistics Netherlands equivalence scale.

In terms of social capital, the survey measures the frequency of contact with the inner circle of family, friends and neighbours. This indicates a person’s strong ties: important others with whom one is emotionally connected through birth, affinity or physical proximity, and who are often considered to be more homogeneous in their resources than a person’s out-group [260]. In addition, the survey allows us to assess the size of the core discussion network [261–263], through a measure of the number of people with whom personal matters can be discussed.

Finally, respondents were asked whether they personally knew individuals with great influence or substantial resources in various domains, a simplified version of the ‘position generator’ [264–266]. We assessed their access to five higher positions: a mayor or member of parliament; a doctor or lawyer; a company director with at least ten employees; a high-ranking civil servant (e.g. municipal secretary or director of a ministry); and a professional musician, artist or writer.

The physical aspect of person capital was gauged by asking about the repondents’ ability to climb stairs and their subjective health. These are common indicators of impairment in performing basic activities of daily living (ADL) and of people’s general health status [267–269]. The survey measured mental capital using validated items on self-confidence, self-image and depression [270–272]. For aesthetic capital, respondents were asked to rate their own appearance and how they thought others would view them. These two generic questions were newly developed for the ‘Disparities in the Netherlands’ survey; they were phrased in a cautious and neutral way, avoiding words such as ‘beauty’ and ‘ugliness’ [273]. The questionnaire also asked people about their height and weight. This was used to calculate their Body Mass Index, which is linked to physical and mental health as well as attractiveness [274–278]. BMI has therefore been treated as a separate hybrid indicator of person capital. We consider all capital indicators to be causal-formative measures: they do not reflect an underlying resource concept, but ‘produce’ the forms of capital. Respondents’ scores on the various measures may correspond, but it is also possible that the indicators capture different resource aspects and therefore show only weak or even negative correlations [279–281].

To examine our second research question, which concerns the relationship of resource disparities and other forms of inequality, we used administrative data from Statistics Netherlands on age, gender, household composition and ethnic background. Labour relations, religion and voting preferences were measured through the questionnaire.

With regard to the third research question, ViN’14 contains several indicators of how people perceive the world (how they view society, the groups they identify with, their trust in others), what they value and strive for, and their well-being (cf. S2 Text). The respondents’ subjective social location was assessed using a standard instrument in which they are asked to position themselves on a ladder running from the bottom to the top of society [282, 283]. The questionnaire recorded whether respondents perceived friction between eight pairs of antagonistic groups, such as rich vs. poor, young vs. old, etc. This was derived from similar questions posed earlier in the *International Social Survey Programme* [237, 282], and resulted in a reliable social friction scale. Societal optimism versus pessimism was measured through the question ‘Do you think things in the Netherlands are generally going in the right or wrong direction?’ [241]. A scale of contentment on social issues [284] was based on items relating to the deficiency of social protection; aversion to cultural differences between natives and migrants; feelings of political abandonment; appraisal of the Dutch power elite; and opposition to further EU integration. Social identification theoretically may occur because people are in similar circumstances, share certain convictions, or emotionally care about the social status of a group [285–287]. In the questionnaire ‘Disparities in the Netherlands’, respondents were asked to what extent they felt themselves to belong to groups that tend to have a great deal of certain types of capital. Exploratory analyses resulted in two scales. The first measures identification with rich, influential and highly educated people, the second identification with young, native Dutch and attractive people. The statement ‘Most people can be trusted’ was used to operationalise trust in others. With respect to personal values, ViN’14 included eight items from the Portrait Values Questionnaire [288–290]; these were condensed into three subscales. In terms of well-being, the survey contained a single-item measure of the respondent’s satisfaction with life and a common question about their ability to make ends meet [291, 292].

### 3.5. Statistical methods

Our first research question was investigated by conducting a latent class analysis (LCA) of the variables listed in Table 1, using the Mplus software package. This technique identifies a limited number of discrete latent classes from observed variables and has several advantages over traditional approaches, such as hierarchical or k-means clustering [293–296]. It is model-based and takes measurement error and missing values into account. In addition, the LCA approach is not deterministic: for each case the probability of belonging to the different latent classes is calculated, rather than assigning it to a particular latent class. Finally, LCA provides a formal statistical test of the optimal number of latent classes, based on empirical fit measures. Unlike Savage *et al*. in their analysis of the British class structure, we employ LCA rather than latent profile analysis because not all of our indicators are continuous (cf. Table 1). In the estimation procedure, the capital indicators were treated as ordered categorical variables [185, 297].

To answer our second and third research questions, we employ nonlinear principal components analysis (nPCA), as available in SPSS 29.0’s CatPCA procedure. The method can be regarded as a variant of multiple correspondence analysis (MCA) and classical PCA [298–301]. It identifies the main underlying components of the multivariate association between variables and depicts their categories in low-dimensional space. This is achieved through a process of optimal quantification, in which categories are assigned numerical values in such a way as to maximise the variance accounted for by the transformed variables. The *vaf*-measure indicates the amount of information retained in the limited number of dimensions [302, 303]. Categories that frequently co-occur among respondents are positioned close together in low-dimensional space. If they rarely do, they are placed far apart. This is also the case within variables: if certain scaled categories of a given variable are close together, their members will have similar profiles on the remaining variables, whereas if they are far apart, these patterns will diverge considerably. In MCA, each category can be positioned anywhere in space (multiple nominal scaling); nPCA allows us to impose additional restrictions, e.g. that categories should be quantified on a straight line. When all the variables are treated as numerical (interval or ratio level), the results are the same as those obtained by classical PCA.

## 4. Results

The three research questions raised earlier are addressed in separate subsections below. First, we present our findings on the contemporary class structure in the Netherlands; then, its empirical links with other social distinctions; and finally, its relationship with socio-political views, subjective well-being, and personal values.

### 4.1. Capital groups in the Netherlands

**Fig 1:**
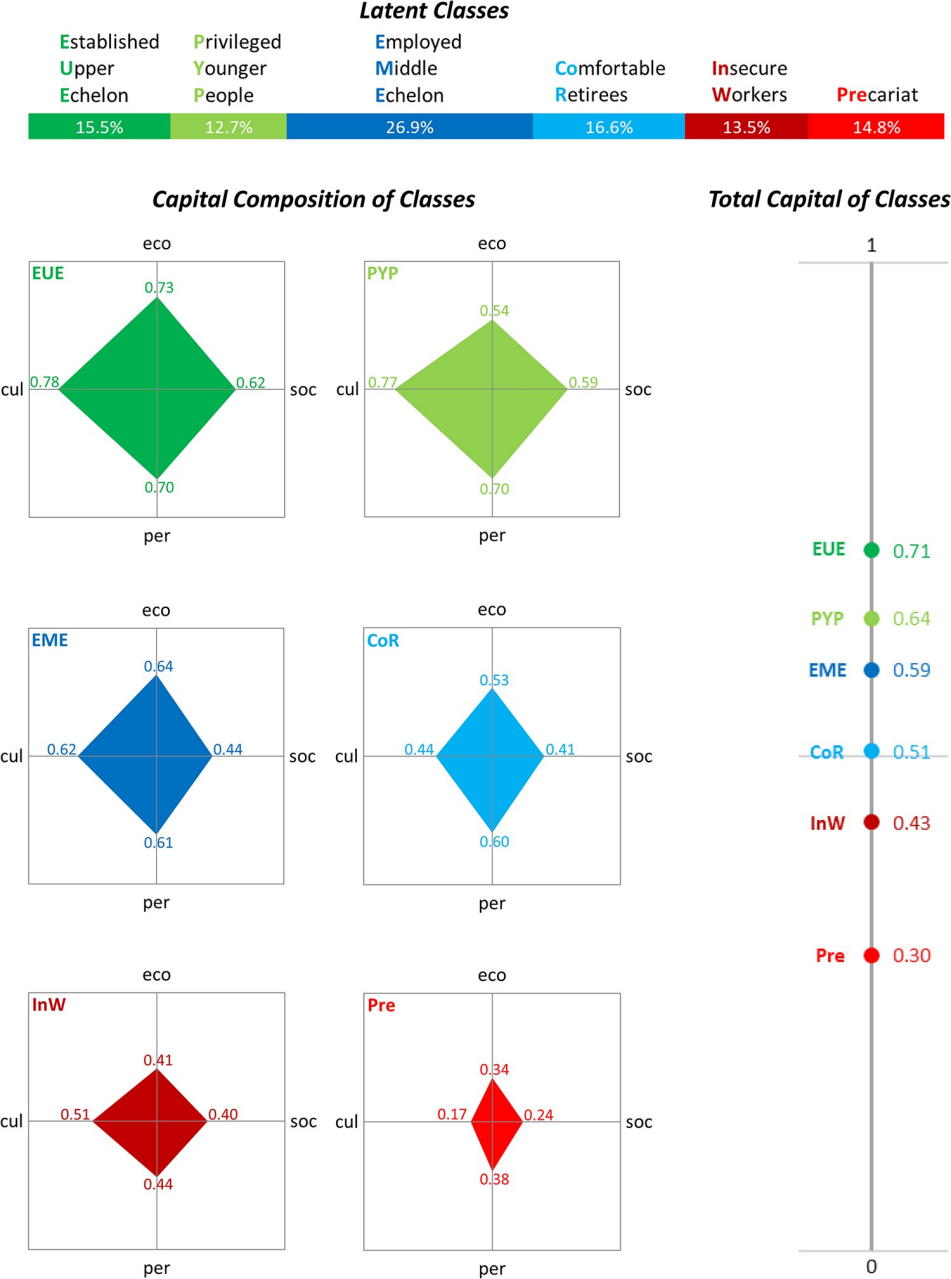
Latent classes in the adult Dutch population, based on four types of capital. All capital scores are on a scale running from zero to one eco = economic capital; cul = cultural capital; soc = social capital; per = person capital.

In the LCA procedure, the Bayesian Information Criterion attains its lowest value when six latent classes are specified (see S1 Table). This model had a good entropy and allowed a sociologically meaningful interpretation. According to the Akaike Information Criterion, a specification with seven classes would also be possible. However, this measure is less suitable for large-N studies such as ours and is more susceptible to overfitting to a specific sample, which could make it harder to replicate outcomes [304]; and a seven-class analysis did not produce any additional substantive insights. Fig 1 shows the results of the LCA with six latent classes: their share in the population, the score of each latent class on the four types of capital, and their total capital. All capital scores were normalised with linear aggregation on scales running from zero to one [305, 306]. The multidimensional nature of the analysis implies that different groups may score quite similarly on a particular type of resource or on total capital. The fact that they end up in different latent classes reflects diverging combinations on the 15 underlying indicators (see the scores listed as S2A Table). The six groups are discussed in more detail below.

### The established upper echelon

Those in latent class 1 have a lot of each of the capital variants and therefore the most total capital (0.71 on a scale from zero to one). Their level of education is high, and they are at the top in terms of disposable income and liquid assets. They generally own their homes and often have substantial equity in these houses. As a corollary, these people enjoy the most luxurious life styles and come second on the other aspects of cultural capital (digital literacy and English language skills). Their social and instrumental networks are large, they have the highest level of mental capital, and only one capital group scores better on physical health, attractiveness and body mass index. This latent class can be characterised as the established upper echelon; it comprises 15.5% of the adult population. Of the top 1% of the standardised income distribution, 91% belong to this group. On the other hand, this class also contains quite a number of persons with slightly lower incomes who score highly on other forms of economic and non-economic capital. The first latent class seems too large to regard the entire group as a ‘power elite’ [307]. The survey, however, does not allow us to assess how many members of the established upper echelon hold top positions in large corporations, politics and public administration.

### The privileged younger people

A second group also has a high level of total capital (0.64), but turns out to be much younger on average. These privileged younger people have fewer economic resources than the established upper echelon: although they are highly educated and tend to be in work, their income and liquid assets are still limited. They often rent their homes; if they own it they have little or negative equity. On the other forms of capital, privileged younger people are well-off. In terms of person capital they score best on BMI, physical health and aesthetic resources, although they lag somewhat on mental health. They have the largest network to discuss personal matters with, but are less likely to know persons in influential professions than the established upper echelon, and they also have slightly fewer contacts with family, friends and neighbours. The cultural capital of this group includes the best ICT skills and command of English, and they rank second in life style indicators. Privileged younger people make up 12.7% of the adult population.

### The employed middle echelon

The third latent class is by far the largest group (26.9%) and has a total capital score of 0.59. Its economic resources exceed those of the privileged younger people. Almost all members of this group are in waged employment and it ranks second in terms of disposable income, reflecting the very high share of dual earners (with children) among them. They are also well educated, but generally less so than the established upper echelon and privileged younger people. Their liquid assets, however, are limited, and they often live in owner-occupied housing with little or negative equity. Cultural and person capital is fairly average, although they score quite high on digital skills, and rather low on aesthetic capital. On social capital this ‘employed middle echelon’ lags behind, particularly because they know few individuals with influential occupations.

### The comfortable retirees

The fourth latent class lies just above the middle of the total capital scale (0.51). They have less economic capital than the employed middle echelon, but more than privileged younger people. This group includes many pensioners and early retirees. On average, they have lower levels of education, but fairly good incomes, substantial liquid assets and high levels of equity in their homes (often bought at a good time and now largely mortgage-free). While these comfortable retirees have rather luxurious life styles, their cultural capital is reduced by their limited digital skills and poor command of English. Their core discussion network is smaller than that of the previous three groups and, like the employed middle echelon, their acquaintance with individuals in influential positions is limited. With regard to person capital there are deficits in physical health, but this group scores rather well in terms of mental and aesthetic resources.

### The insecure workers

The fifth latent class is further down the scale of total capital (0.43). It has meagre economic resources, as a result of low income, indebtedness and scarce liquid assets. They have a weak position in the labour market: the employed have the highest share of temporary contracts, and many are jobless. The members of this group mostly live in rented accommodation; homeowners are few and often in negative equity. In terms of education, however, they do not rank particularly low. The same applies to their cultural capital: a less luxurious life style is partly compensated for by their digital skills and proficiency in English, which exceed those of the comfortable retirees. Their social capital is not extremely low either, but person capital lags behind the four higher-ranked groups, particularly on mental and aesthetic resources, where they occupy the lowest position. Given the combination of an uncertain labour market position, low self-confidence, poor self-image and high levels of depression, the term ‘insecure workers’ describes this class, which makes up 13.5% of the population over the age of eighteen.

### The precariat

The final latent class is the mirror image of the established upper echelon. Its members have few resources of any kind, and their total capital is therefore very modest (0.30). They have a low level of education and limited income, living mainly on benefits or a modest pension. These people are generally in debt or have low levels of liquid assets, and it is rare for them to own their homes. Their life styles are the least luxurious of all the capital groups, they have very few digital skills and show the poorest command of English. They also come last in all forms of social capital, most notably so in their almost complete lack of access to persons in positions of influence. Physically, they are the least healthy and often overweight. Due to their general lack of recources, this group can be described as the precariat, in line with Savage *et al*. [185]. They account for 14.8% of the adult Dutch population.

### A social class structure

In this analysis, we have identified six capital groups that can be ranked according to their total resources. Each group has a distinctive mix of the four types of capital. We have previously argued that class antagonisms are theoretically more pervasive when there is less fragmentation, more multiple correspondence, greater scope and more stability over time. Using these criteria, we may conclude that the nature of the resource disparities between the six groups indicates a clear social class structure. Although our results do not show a simple juxtaposition of ‘haves’ and ‘have-nots’, the number of groups is smaller than in traditional class schemes, such as the widely used later version of the Erikson-Goldthorpe-Portocarero typology or Wright’s neo-Marxist classification, which contain eleven and twelve classes respectively [308, 309]. It corresponds to the degree of fragmentation of the Gilbert-Kahl model, which divides modern US society into six social classes based on people’s education, occupation and income [310, 311]. The consistency in the positions on the four types of capital is also fairly high: the entropy of the latent class model is 0.76, indicating substantial multiple correspondence (cf. S1 Table). The difference in total capital between the established upper echelon and the precariat covers more than 40% of the underlying scale, so the scope between the top and bottom groups is considerable. Social distance is also evident in the patterns of residential separation of the capital groups (see S3 Text). As our data are cross-sectional, we do not know whether the respondents are in the same resource class as their parents or remain so over the life course, and we cannot assess the stability criterion in detail. However, the information we do possess suggests that the picture may be consistent over time. In the six classes we find limited educational and occupational mobility between generations, while educational homogamy is substantial. This is particularly evident in the two extreme classes, the established upper echelon and the precariat (cf. S4 Text).

### 4.2. Social classes and other distinctions

Nonlinear PCA was conducted in order to assess how the six social classes are related to other social distinctions. These pertain to a number of demographic variables (age, ethnic background, gender, household composition); and whether or not people are religious (including non-Christian faiths and non-practising believers). We also take into account their voting intentions, on the materialist assumption that these will be a function of their objective social position. For labour relations we consider two variables. The first indicates whether employees had a permanent or a temporary contract. The large number of missing observations, resulting from self-employed and inactive persons, have been scaled passively and do not affect the quantifications. Furthermore, we include the Erikson-Goldthorpe-Portocarero typology in the analysis. This will allow us to explore the similarities and differences between our contemporary class structure and a common division into ‘Big Classes’, which reflect the authority and market position of occupational groups in late industrial society. To the eleven EGP classes we add a category for those who have never had paid work, such as certain housewives and early disabled persons. For pensioners and benefit recipients, the last known type of work was used to determine their EGP class. The occupational status of students could often not be established; these cases were handled as missing.

All variables were treated as active in nPCA, as we are interested in the multivariate contingency with the class structure in low-dimensional space. Single spline-nominal scaling was used for age, occupational class, ethnic origin, religiosity and gender; the remaining variables were treated as multiple-nominal. This specification of the analysis levels implies that if the scaled categories retain their original order, it is due to empirical associations in our dataset.

In a preliminary analysis with oblimin rotation, a very low correlation was found between the first two axes (*r* = 0.019). We therefore opted for orthogonal varimax rotation, which allows a more straightforward interpretation of the outcomes. Cronbach’s α is rather high (0.80); in nPCA this measure indicates the global fit of the solution [312: 56]. The two dimensions jointly account for 38.9% of the total variance, and are almost equally important (*vaf* DI = 2.8; DII = 2.2). Adding further dimensions does not lead to new substantive insights. Without the constraint that five variables should be scaled on a single vector through the origin, the total variance accounted for would not be much higher (3.6 instead of 3.5). This implies that the categories of these characteristics are already almost on a straight line when a more lenient multiple-nominal scaling level would be specified. Bootstrapping with 1000 random samples shows that the eigenvalues obtained are within the 95% confidence intervals, and that the variance accounted for by each of the variables differs very little from their bootstrap mean (maximum deviation = 0.009).

### Interpretation of the dimensions

**Fig 2:**
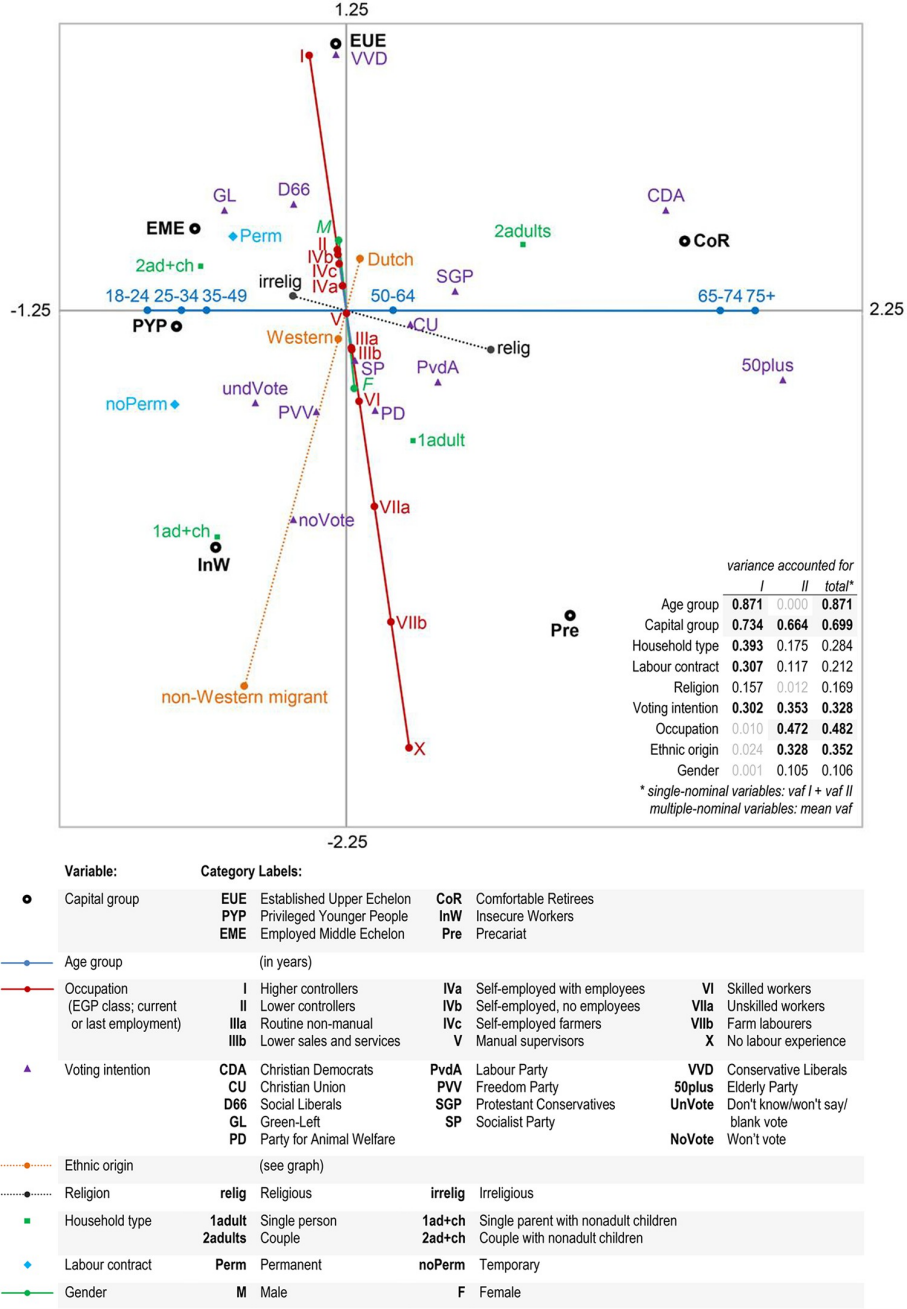
Capital groups, socio-demographic traits and voting intentions (rotated nPCA category coordinates).

The first dimension of Fig 2 reflects in particular distinctions by age and capital group, with contributions to the variance accounted for of 0.87 and 0.73 respectively. These age/capital differences are related to the presence of minor children in the household (*vaf* = 0.40), voting intentions and the type of labour contract (*vaf* = 0.30–0.31), and being religious or not (*vaf* = 0.15). Distinctions by gender, occupational class and ethnic origin hardly matter here (*vaf* = 0.00–0.02). The dominant age variable shows contrasts between those over sixty-five (rather extreme positive scores on the horizontal axis), a middle-aged group (slightly above the mean score of zero), and those below fifty (negative scores that are closely scaled). The six classes are ranked according to their mean age. Privileged younger people, the employed middle echelon and the insecure workers are comparatively young (mean = 35–40 years) and therefore have negative scores, while the older precariat and the comfortable retirees (mean = 63–66 years) end up at the positive side. The established upper echelon (mean = 49 years) falls in the middle. Respondents in households without minor children are generally older, as are employees with a permanent labour contract and religious persons. By voting intention, there is a stark contrast between the older electorates of the Christian Democrats (CDA) and the Elderly Party (50+), and the on average younger supporters of the Green Left plus the Social Liberals (GL, D66).

The substantial number of swing voters and non-voters were also comparatively young. The clustering on the positive side of the horizontal axis indicates that a comparatively large share of the comfortable retirees intended to vote for CDA or 50+ (combined with limited support for GL, and few non-voters and don’t knows). This dimension can be referred to as *age-based capital disparities and related factors*, including differences in voting patterns by age.

The vertical axis shows contrasts between more and less resourceful capital groups (*vaf* = 0.66), which partly coincide with different occupational classes (*vaf* = 0.47). These dominant variables are combined with class-related differences in voting intention and ethnic origin (*vaf* = 0.33–0.35); and to a lesser extent with contrasts in household type (having or not having a partner), type of contract, and gender (*vaf* = 0.11–0.18). Age and religion are of little importance (*vaf* = 0.00–0.01). The ordering of our six classes is rather similar to the group differences in total capital in Fig 1, although the privileged younger people are below the employed middle echelon and the comfortable retirees. It partly corresponds with the EGP occupational class ranking; and native Dutch, couples, employees on a permanent contract and men generally reach higher positions than their counterparts. These categories therefore tend to belong to the higher classes in terms of capital and occupation, to be relatively uncommon in the lower ones, or both. With regard to voting intentions, a positive score—indicating an overrepresentation of resourceful groups, higher EGP classes, native Dutch etc.—is obtained by supporters of the Conservative Liberals (VVD), followed at some distance by those backing the Christian Democrats, Green-Left and the Social Liberals (CDA/GL/D66). Non-voters scored lowest. Respondents who supported parties that focused on the economic interests of disadvantaged and non-working persons in 2014—the Labour, Socialist, Elderly and Freedom parties (PvdA/SP/50plus/PVV)—also ended up on the negative side, as did people who had not yet decided how to vote. A remarkably homogeneous cluster can be seen at the top of Fig 2. Membership of the established upper echelon often coincides with belonging to the occupational class of higher controllers and intending to vote for the Conservative Liberals. That particular combination comprises 13% of the most resourceful social class, and 2% of the entire sample. Three-quarters of these upper-class VVD supporters are men, and hardly any of them have a non-Western ethnic background. Altogether, this dimension may be regarded as a summary measure of the *general social hierarchy*, and its translation into voting preferences.

### The age basis of resource disparities

On the first dimension there is a strong correlation between age and capital group (*r* = 0.66). This contingency is partly driven by the age distributions of the comfortable retirees and the precariat, who attain the highest scores (see S1 Fig). Individuals over sixty-five are strongly overrepresented in these capital groups, while young persons are rare. The precariat includes the largest share of people over seventy-five, but the comfortable retirees are on average the oldest. This is due to their larger contingent in the 65–74 age bracket and fewer people under fifty. Privileged younger people, the insecure workers and the employed middle echelon also contribute substantially to the high correlation. These classes contain hardly any members over sixty-five. In addition, the majority of the privileged younger people are under thirty-five, while the 50–64 age bracket is underrepresented among them, resulting in the lowest mean age. The most common age category of the employed middle echelon and the insecure workers (35–49) is higher, but here the younger age groups are also overrepresented. In the employed middle echelon, this reflects a strong presence of young adults who have not yet left the parental home: 14% of this capital group consists of co-residing children aged eighteen to twenty-four. Members of the established upper echelon are mostly in the two age categories in the middle. People below thirty-five are underrepresented in this class. While it includes a fair share of sixty-five- to seventy-four-years-olds, the highest age category is almost empty.

On the horizontal axis of Fig 2, the correlations of the social class structure with household composition, the type of labour contract, voting intention and religion (*r =* 0.03–0.30) are generally weaker than the correlations of age with these variables (*r =* 0.13–0.45); and all these contingencies are by no means perfect. The mean values of the six capital groups on the transformed variables (see S3 Table) show that their position on this dimension is mainly determined by their age composition. However, the negative score of the privileged younger people is amplified by the fact that they are often non-religious and intend to vote for parties with a young following. The same reinforcement applies to the employed middle echelon, but their position also reflects the large share of couples with minor children. Among the insecure workers, household composition likewise pushes the score down. The extreme position of the comfortable retirees and the precariat reflects, in addition to a large share of elderly persons, a higher level of religiosity and the absence of minor children. In the case of the comfortable retirees, their positive score is compounded by age-related voting intentions.

The strong link we find between age and the resources of the six capital groups may reflect the impact of historical events on various age groups (cohort effects) or processes of capital (dis)accumulation over individual life courses (ageing effects). Since we have only one point in time, by definition we will not find any period effects. Empirically, many resources show a rather steady downward trend: for nine indicators, capital decreases with age (see S5 Text). This is partly offset by the fact that other resources (liquid assets, housing wealth, mental health) tend to increase with age, or continue to do so until retirement age (household income, the life style score). On balance, total capital tends to decline somewhat as people get older. This suggests a complex relationship between resource levels, cohort membership and ageing over the life course.

### Capital groups, occupational class and other forms of hierarchy

On the vertical axis of Fig 2, the correlation of capital groups with occupational class (0.50 for all respondents, 0.48 for those currently employed) is substantial, but weaker than their association with age on the horizontal one. Several occupational classes are not ranked as assumed in the EGP typology, and the concentrations in the six capital groups are not entirely consistent (see S6 Text). This suggests that the resource differences between the six capital groups cannot be reduced to the occupation-based Big Classes of the industrial era. In contemporary Dutch society, the EGP class scheme does not fully capture the impact of non-occupational forms of capital on the distribution of life chances.

The correlations of ethnic origin, presence of children, type of labour contract and voting intention with the capital group are not particularly strong (0.18–0.33), although they mostly exceed those with the EGP typology (0.08–0.28). For gender, however, the association with occupational class predominates (cf. S10). Thus, these hierarchical distinctions also partially coincide with the social class structure.

The ranking of the six capital groups on the vertical axis reflects their scoring pattern on all other variables (cf. S3 Table). The established upper echelon occupies the top position due to a comparatively large share of higher occupations, few persons of non-Western ethnic origin, often having a partner, and an inclination to vote for conservative or social-liberal parties. Insecure workers and the precariat end up on the other side owing to a large share of people with low jobs or no work experience, and an overrepresentation of non-Western migrants–although they are not a majority even in the two groups with the least capital. The low position of these capital groups is reinforced by the fact that a disproportionate number of people do not have a partner, do not intend to vote, and are on temporary contracts. The privileged younger people are second best in terms of occupational class, but their score on the vertical dimension of Fig 2 is depressed by a relatively high share of non-Western migrants, and by the fact that they often are single and on temporary contracts. The opposite occurs among the employed middle echelon and the comfortable retirees: their scores are boosted by the fact that they are mostly of native origin and relatively often have a partner, and because the employees among them tend to have a permanent contract. Consequently, despite their rather average scores on occupational class, these two capital groups end up higher than the privileged younger people.

### Structural class disparities coincide with other forms of social hierarchy and age differences

In response to our second research question, we found that the structural class differences identified in the previous section go hand in hand with other distinctions that span two main dimensions. On one dimension, characteristics emerge that mirror the general social hierarchy in contemporary Dutch society. Resource disparities between higher and lower social classes coincide to some extent with differences by (former) occupation, ethnic origin, type of employment contract, and gender. This also translates into voting intentions that reflect the present social hierarchy. The other main dimension shows differences between age groups. These reflect a complicated relationship between resource levels, cohort membership, and ageing during the life course. Several other age-related differences (the presence of minor children in the household; working on a temporary contract; being religious; age-related voting) also appear on this axis.

### 4.3. Socio-political views, well-being and values in the class structure

To answer the third research question, we conducted yet another nonlinear PCA. This aimed to examine the extent to which social classes differ on seven aspects of socio-political views: the location people assign to themselves on the social ladder; the social frictions they perceive; two forms of self-identification with social groups; (lack of) optimism about Dutch society; (dis)contentment with specific social issues; and trust in others. We also included two indicators of well-being: people’s satisfaction with life and their ability to make ends meet. Finally, we examined the extent to which our respondents endorsed the personal values of self-enhancement and hedonism; two weaker Schwartz scales (cf. S2) were discarded. All these variables were already ranked in their original format and were therefore treated as spline-ordinal in nPCA. As in the previous section, the analysis level of the capital group variable was set to multiple-nominal. Missing values were passively scaled and therefore do not affect the outcomes.

We present a solution with two unrotated dimensions; the differences between the six classes are well interpretable on the original principal components. Cronbach’s alpha is high (0.88), and the two dimensions jointly account for 44.4% of the variance. The first dimension is much more important than the second (*vaf* DI = 3.7; DII = 1.6), and no insights are gained by adding a third dimension. The total variance accounted for is not much reduced (4.9 instead of 5.1) by the constraint that ten of the eleven variables must lie on a straight line through the origin, while maintaining the order of their categories. Bootstrapping (1000 random samples) shows that the eigenvalues are within the 95% confidence intervals. Moreover, for each individual variable, the *vaf* is hardly different from the bootstrap mean: the maximum deviation is 0.04.

### Interpretation of the dimensions

**Fig 3:**
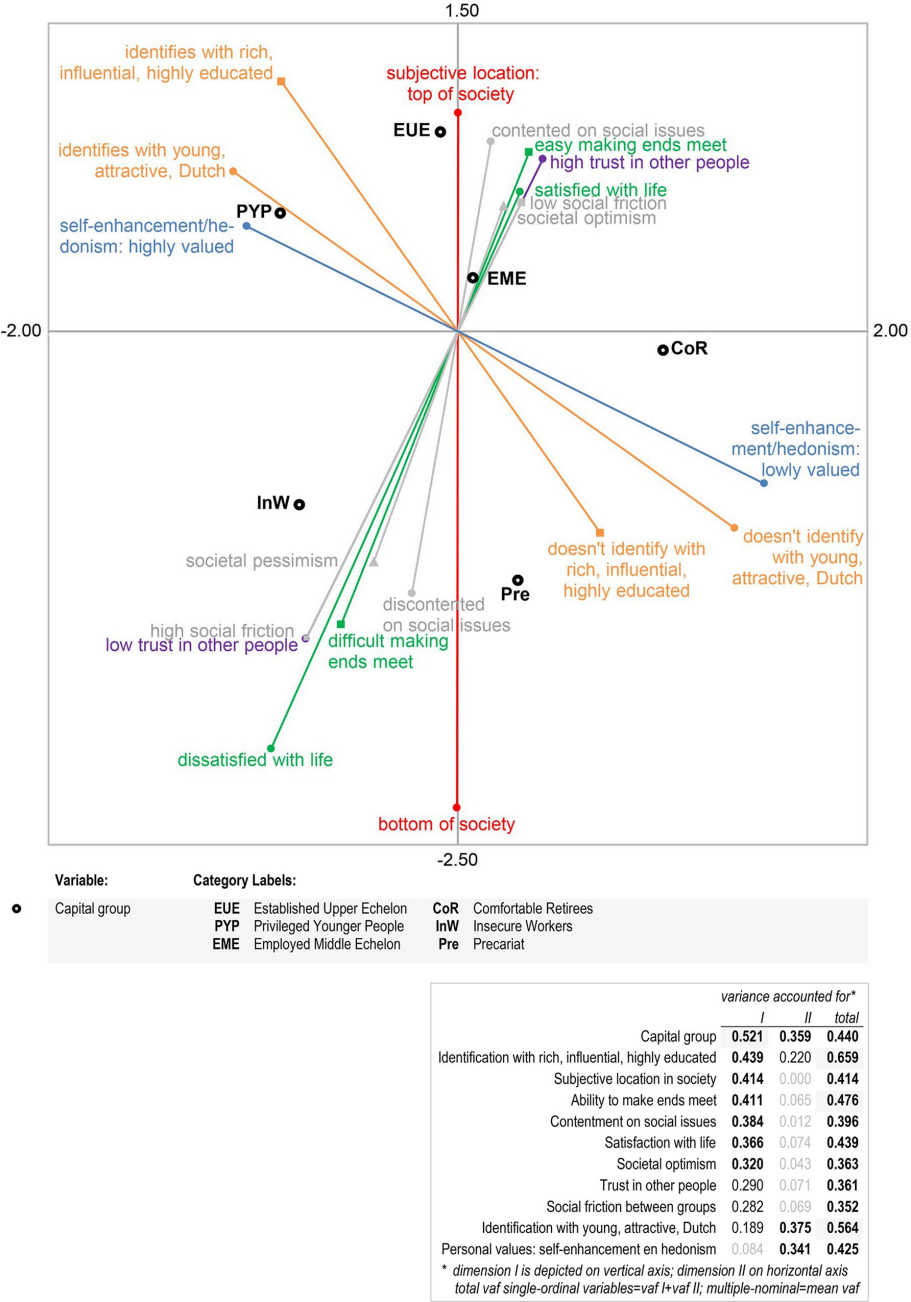
Capital groups, socio-political views, well-being and personal values (nPCA category coordinates).

For the sake of comparability with the graph in the previous section, the two axes of Fig 3 have been inverted. All variables contribute to the first dimension (shown vertically), with component loadings generally between 0.53 and 0.66, but lower for identification with young/attractive/Dutch people (0.43) and the self-enhancement/hedonism scale (0.29).

The social class variable reaches the highest *vaf* on the vertical axis. The six capital groups are ranked according to their total resources, with a fairly large gap between the precariat plus precarious workers and the four higher social classes. This reflects the fact that many subjective variables were already ordered by resource levels before scaling. For subjective location on the social ladder, societal optimism, social friction, contentment on social issues and identification with rich/influential/highly educated people, the differences in the mean scores correspond to the ranking of classes by total capital in Fig 1. On the other variables, the maximum deviation is one position. A one-way ANOVA with post hoc contrasts shows that on most indicators, the precariat and the insecure workers form a homogeneous cluster with scores well below the mean. This accounts for the large distance between these two classes and the other four. All in all, the first principal component can be characterised as a general measure of the *subjective class hierarchy* relating to socio-political views, well-being and personal values.

The contrasts that remain on the second principal component (shown horizontally) are limited. These are mainly driven by differences in self-identification and personal values, with component loadings ranging from 0.47 to 0.61; and by social class, with the second highest *vaf*. On the horizontal axis of Fig 3 we see a contrast between privileged younger people and insecure workers on the one hand, and comfortable retirees on the other. This occurs because the two younger capital groups identify more strongly with the young/attractive/Dutch and value self-enhancement and hedonism more than their resources would suggest. In addition, comfortable retirees identify less with the rich, influential and powerful than their total capital may lead one to expect. The second dimension thus measures certain *non-hierarchical differences in self-identification and personal values*. It is worth noting that the six capital groups are not clearly ranked by their average age, as was the case on the horizontal dimension of Fig 2. The employed middle echelon is quite distant from the two other social classes that contain few over-50s; and the elderly precariat has a much lower score than the comfortable retirees.

### Profiles and correlations by social class

On average, members of the established upper echelon place themselves highest in society, see the least social friction, are most contented on social issues, identify most strongly with the rich/influential/highly educated, and are most optimistic about society. They are also the happiest with their own lives, have the fewest problems making ends meet and trust others the most. The established upper echelon comes second in terms of identification with the young/attractive/Dutch and the values of self-enhancement and hedonism. On these variables, privileged younger people achieve the highest mean score. Otherwise this capital group are second—except for the ability to make ends meet, where they end up below the employed middle echelon. That social class is usually in third place. Their experience of doing well financially is partly offset by the fact that they trust other people less than the comfortable retirees. This older capital group typically ranks fourth on socio-political views and well-being, but fifth on the two identification variables and the personal values to which they adhere. Insecure workers often occupy fifth place, but not on identification with the young/attractive/Dutch and on the value they place on self-enhancement/hedonism (fourth)–although here too they score below the general mean. On the downside, insecure workers report the lowest ability to make ends meet, have the least trust in others, and the greatest dissatisfaction with their own lives. The pattern observed among the precariat is the mirror image of the established upper class: they score much lower than average in every respect. Members of this capital group position themselves at the bottom of society, experience the most social friction, identify least with advantaged groups, are the most pessimistic about the direction of Dutch society, and are the most discontented with social issues. They also place the least value on self-enhancement and hedonism. On the three remaining characteristics, the precariat comes fifth, slightly above the insecure workers.

These patterns yield substantial correlations with social class structure for five variables: subjective location, contentment on social issues, life satisfaction, making ends meet, and identification with the rich, influential and highly educated (*r =* 0.33–0.44). The associations with the four remaining characteristics are somewhat weaker (*r =* 0.19–0.28). An additional analysis examined the links with the objective disparities from the previous section (cf. S7 Text). This revealed, among other things, that subjective experiences are more strongly associated with the six capital groups than with the EGP occupational classes, age and the ‘age-based capital disparities’ dimension. Correlations with social class are higher or equal to those with the ‘general social hierarchy’ dimension, depending on the subjective characteristics considered.

### Structural class disparities are linked to socio-political views, well-being and personal values

Regarding our third research question, we observed a consistent relationship between the contemporary class structure and the subjective ideas and experiences of the respondents. The ranking of the six social classes in terms of resources is clearly reflected in differences in socio-political views, well-being and certain value orientations. When classes have few resources, they hold more negative views about politics, society and their own position in it. Furthermore, they are less committed to the values of ‘self-enhancement’ and ‘hedonism’ than classes with a lot of resources, while their well-being is lower. These findings indicate that disparities in the four types of capital are connected to social cohesion problems. Views on the role of government and the common good may differ considerably between classes; and some capital groups are likely to seek social closure at the expense of others. It is also important to recognise that people’s subjective ideas and experiences are more closely related to the contemporary resource-based class structure than to traditional occupational distinctions or age.

## 5. Conclusions and discussion

The key findings of this study imply an affirmative answer to the three research questions posed in section 3.2. Through a dedicated survey on people’s economic, social, cultural and person capital, linked to national register data, we first examined the Dutch class structure in 2014. Using latent class analysis (LCA), we identified six groups. The established upper echelon (15.5% of the adult population) has the most capital, followed by the privileged younger people (12.7%), the employed middle echelon (26.9%) and the comfortable retirees (16.6%). Total capital is lowest among the insecure workers (13.5%) and the precariat (14.8%). Each group has a distinct mix of the four types of resources. We consider this to be a pervasive class structure because the division into capital groups fulfils certain theoretical conditions: limited fragmentation, substantial multiple correspondence, and a large scope between the top and bottom classes.

Secondly, we found that this structure is connected to other hierarchical distinctions in contemporary Dutch society, based on occupation, ethnic origin, type of employment contract and gender. In addition, each class has a specific age profile. The privileged younger people have the largest share of persons under the age of thirty-five. Insecure workers and the employed middle echelon are slightly older on average, but mostly under fifty. The established upper echelon has a large contingent of 50–64 year olds, while the comfortable retirees and the precariat are mainly made up of older persons. This class-age nexus reflects the importance of cohort membership and ageing processes in the evolution of resources. It is echoed in other age-related social class differences (having minor children or a permanent contract, being religious). The voting intentions of the six classes vary according to their position in the social hierarchy and their mean age.

Finally, we observed a consistent relationship between the contemporary class structure and people’s subjective ideas and experiences. The ranking of six social classes in terms of total resources clearly recurs in our respondents’ socio-political views, well-being and certain value orientations. Classes with little capital generally rate politics, society and their own social position more negatively. They also value self-enhancement and hedonism less than today’s upper classes, and report lower levels of well-being. This suggests that multidimensional resource differences between classes are intertwined with issues of social cohesion.

### Theoretical considerations

From a theoretical point of view (cf. section 2), our findings demonstrate the added value of a multidimensional capital approach. As in a previous British study, it leads to the identification of a new and complex layered structure. Like these authors, we find two extreme groups where capital (dis)accumulates across the board, with in between ‘a patchwork of several other classes, all of which have their own distinctive mixes of capital’ [186:53]. Yet the number of classes in the Netherlands is smaller (six rather than seven), and the capital groups are not entirely comparable. This may reflect a real divergence between the Dutch and British class structures, possibly related to institutional differences (see S1 Text). It could also be due to our introduction of health and attractiveness as a fourth type of capital, and our more comprehensive measurement of economic, social and cultural resources. Finally, differences in data sampling and sources may have played a role. Our respondents were selected at random rather than by quota or convenience sampling; and the income and wealth indicators are based on reliable official registers instead of self-reports.

Through our analysis we still found distinct social classes, but these are different from the occupation-based Big Classes of the late industrial era (such as the Erikson-Goldthorpe-Portocarero typology, cf. section 4.2). Our six-class hierarchy does not preclude the existence of micro-classes that are reproduced from one generation to the next; but to assess their relevance within the post-industrial class structure, the various generic and job-related resources of small occupational groups should be measured directly and comprehensively. More generally, it is of theoretical significance that the class disparities identified here are partly due to differences in economic resources; however, the divergent positions in the contemporary Dutch class structure cannot be reduced to financial inequality or traditional classifications of socio-economic status. For example, privileged younger people have a lot of capital in many respects, which gives them a relatively high position in society and favourable life chances; yet their income and wealth are limited for the time being. Economic resources are not irrelevant, but they are interconnected with the other three forms of capital–and these may be decisive for the emergence and evolution of post-industrial class divisions.

Our capital approach sheds some light on the emerging inequalities we discussed in chapter 2. The fact that 28% of the Dutch population has few resources is consistent with notions of the existence of a post-industrial service proletariat, and of benefit recipients and pensioners as constituent elements of the class structure. On the other hand, there is no clear evidence of a polarisation that has squeezed out middle class workers: the employed middle echelon is by far the largest capital group. It is also worth noting that we did not find a distinct class of ‘one-percenters’: individuals whose vast financial resources allow them to shape the organisation of society to their liking. Perhaps this phenomenon is less pronounced in the Netherlands than elsewhere, or perhaps the group is too small and diverse to be captured in our LCA: one per cent of the sample is only 30 respondents. However, it is also possible that this economic power elite is in fact a much smaller group (e.g. the five hundred wealthiest or politically most influential persons, representing merely 0.004% of the adult Dutch population), or that some of them reside elsewhere. Finally, the wealthy elite might not be very different from the slightly less affluent in terms of certain non-financial resources (such as educational attainment or health). Be that as it may, our study indicates that the class structure in the Netherlands encompasses more than a simple division between the top one per cent and the rest of the population.

As for cognitive stratification, we do not have data on our repondents’ intelligence and specific cognitive skills (such as information processing, perception, memory), but only on their level of education. The lower the social class, the lower this is, with one exception: insecure workers were on average slightly more educated than comfortable retirees (see S2A Table). The correlation between educational attainment and total capital is substantial but not perfect (*r* = 0.60), nor is it the only strong link with people’s combined resources (S2B Table). If there is cognitive stratification, one would expect a tight connection between education and all the other types of resources. Yet the correlations with personal and social capital are not pronounced (*r* = 0.24, 0.36), and at the indicator level, we only find strong links of educational attainment with digital and English skills (*r* = 0.47, 0.56). This leads us to conclude that there is an educational gradient in people’s resources, but that we cannot equate class disparities with cognitive stratification. Of course, many types of work have formal educational requirements, and where this is not the case (e.g. political positions) the educational gradient may still play a role [313]. But, as noted above, the class structure today involves more than a link between education and labour market status.

The class disparities found here also contradict the liquefaction thesis of postmodernist theory. Social classes have not disappeared, even if today they are different from the Big Class divisions that were criticised by this school of thought. Nevertheless, one of its core tenets—that individual life styles and identities matter for the allocation of social positions—fits well with our notion of person capital. This concept also incorporates theoretical ideas about health and attractiveness as important resources, with complex underlying mechanisms of selection and causation. In our empirical analyses, person capital indeed proved to contribute to the contemporary class structure.

### Research agenda

The capital approach taken here, combined with other aspects of social hierarchy and subjective experiences and evaluations, provides an interesting starting point for future research on social class. The main challenge ahead is to examine how contemporary forms of capital-based structuration emerge under different social conditions and heterogeneous institutions; which groups of actors are likely to invest more or less in their resources; how they use them to improve their life chances and exercise power; and how this then feeds back into the evolution of the class structure, other forms of hierarchy, and social cohesion. Furthermore, it is important to test the stability and generalisability of our findings by replicating the study in the Netherlands and elsewhere—especially in countries with different institutional regimes, such as the generally smaller Anglo-Saxon systems; the traditionally more universalistic welfare states of Scandinavia, and more selective ones in Western and Southern Europe; post-communist nations; and emerging economies [193, 314].

Our capital indicators can also be improved in a number of ways. We did not have information on the symbolic elements of cultural capital (reputation, titles, awards, fame, language, people’s first and last names). It would be better if indicators of social capital referred not only to the number or frequency of social and professional contacts, but also to the actual resources that networks contain. With regard to person capital, the aesthetic and mental aspects in particular could be measured more comprehensively, through more detailed indicators of individual (un)attractiveness, charm, personality, resilience and so on. It is also worth considering whether specific forms of person capital (or combinations thereof) give people key advantages or disadvantages in society. A direct measurement of certain resources, rather than self-reports, might prove useful, as might an assessment of the role of good fortune and bad luck in achieving different social positions over the life course.

### Policy implications

An analysis of class structure based on four types of resources may open up new avenues for social policy. It is important to tackle the large social class disparities we have identified for three reasons [315]. First, they affect the nature of society and run counter to values such as equality, fairness and solidarity. Second, the class hierarchy is likely to reflect causes that are difficult to justify (unequal distribution of power, biased laws, discrimination, excessive intergenerational transfers). Finally, these class inequalities can have undesirable consequences for society as a whole, such as significant group differences in well-being and political trust, an undermining of social cohesion or low legitimacy of public policies.

In social policy, a purely economic approach of the current class disparities is unlikely to be sufficient. Focusing exclusively on promoting equal opportunities in education, reducing inequalities in the labour market and addressing excessive differences in income and wealth ignores the interconnectedness of resources. Where you fit in, who you know and who you are can then still evoke or maintain class distinctions. Disparities in terms of cultural and social resources, as well as health and attractiveness, therefore also deserve policy attention. A policy of personal responsibility—in the sense that class divisions are expected to diminish automatically if individuals are encouraged to invest in their own resources—is also unlikely to be effective. Such an approach disregards the structural nature of class inequality and can trigger Matthew effects [316–318].

A more promising approach is for governments to address the combination of resource deficits among the lower social classes. This can be achieved, for example, through policies aimed at the poverty and loneliness of the precariat, or the combination of mental vulnerability and job instability of insecure workers. However, such a strategy of ‘levelling up’ multidimensional resource deficits is often less straightforward for older persons, as this group has largely completed the process of capital (dis)accumulation. It also preserves the resource advantages of the higher classes: above the bottom, the social hierarchy is likely to remain intact.

A collective approach with three priorities is, in our view, the best way forward. First, governments should invest in the resources persons need at key life transitions: selection moments during education; entering and leaving the labour market, and the careers in between; household formation; people’s declining physical health as they age. Such a social investment strategy should be combined with a second element: optimal institutional complementarity [319–323]. This means that the objectives, rules and delivery processes of different parts of the public sector—the systems of education, childcare, labour market regulation, social security, health care, pensions, housing, and income and wealth taxation—are developed in a coordinated and integrated way, in order to optimise the allocation of resources throughout people’s lives. Third, the design of the welfare state should be guided by the principle of ‘proportional universalism’ (also known as targeting within universalism) [324–326], an idea that has been gaining ground in the academic debate on social policy in recent years [327–333]. The starting point is Dworkin’s thesis that those in poor health, for example, need more resources to achieve the same as others in society. If unhealthy and healthy persons are granted identical rights, the two groups will have unequal opportunities to achieve a favourable social position [334]. Proportional universalism implies that every citizen is entitled to certain public services and facilities (e.g., education, basic health care) without further conditions. In addition, some groups receive compensation for existing social inequalities in resources, while others are required to make additional contributions. The three-pronged approach we recommend may also partially break the link between class differences, other hierarchical distinctions and people’s subjective ideas and experiences. In this way, social policy can help to make the contemporary class structure and its ramifications less harsh.

## Supporting information

S1 Fig Age profile of capital groups. (PDF)

S1 Text The Netherlands: Developments, institutions and policy-making. (PDF)

S2 Text List of survey items. (PDF)

S3 Text Residential separation of capital groups. (PDF)

S4 Text Educational homogamy and absence of intergenerational mobility. (PDF)

S5 Text Distribution of resources by age. (PDF)

S6 Text Capital groups and occupational classes. (PDF)

S7 Text Correlations of subjective measures with objective disparities and voting intentions. (PDF)

S1 Table Fit measures of the latent class analysis. (PDF)

S2 Table Capital indicators: Mean scores by latent class and correlations. (PDF)

S3 Table Mean scores of capital groups on the transformed variables of Fig 2. Striking image. (PDF)
