# Supplementary material for: A contemporary class structure: Capital disparities in The Netherlands
Source: PLoS One. 2024 Jan 31;19(1):e0296443. doi: 10.1371/journal.pone.0296443 (PMC10830037; doi:10.1371/journal.pone.0296443)

S7 Figure. Age profile of capital groups

S7 Figure. Cumulative age distributions of six capital groups

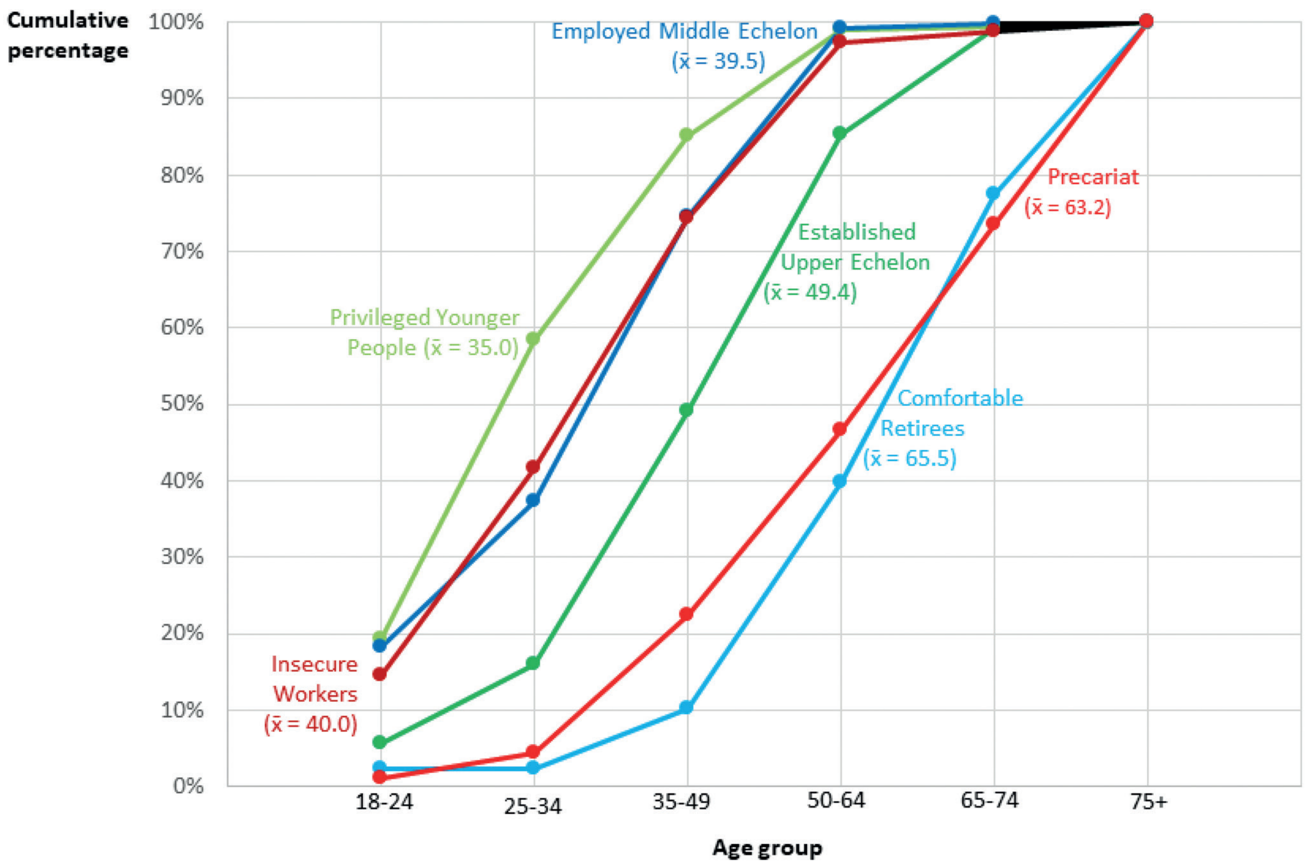

Supplement: S1 Fig — (PDF) [file pone.0296443.s001.pdf]
