# Supplementary material for: A contemporary class structure: Capital disparities in The Netherlands
Source: PLoS One. 2024 Jan 31;19(1):e0296443. doi: 10.1371/journal.pone.0296443 (PMC10830037; doi:10.1371/journal.pone.0296443)
Supplement: S1 Text — (PDF) [file pone.0296443.s002.pdf]

S1 Text. The Netherlands: Developments, institutions and policy-making

The Netherlands is a highly de-industrialised country: on the labour market, the share of people employed in the primary, manufacturing and construction sectors fell from 31% in 1980 to 17% in 2014, while growth occurred mainly in public and business services [1]. The service-oriented Dutch labour market also became 'the first part-time economy in the world' [2]: female participation accelerated after 1985, mostly through permanent part-time contracts offering a high level of social protection. This coincided with the replacement of the dominance of the male breadwinner family by a 'one and a half earner' model. In addition, a comparatively large share of employees are on temporary contracts, partly through employment agencies; and after 2000 the number of self-employed with no staff grew sharply. Atypical and sometimes precarious work has also become more common in the new millennium, for example in the form of zero-hours contracts, platform employment, posted workers and bogus self-employment [3-6].

The Netherlands is one of the most developed economies in the world. Among countries with a population of 100.000 or more, it ranks thirteenth on GDP per capita, second in terms of economic globalisation, and tenth on the Human Development Index; and both businesses and individuals are well connected to the digital economy [7-11]. Income inequality is fairly limited and stable by international standards, and poverty rates are low, especially among pensioners. In-work poverty, however, has increased structurally in the new millennium. Wealth disparities are comparatively high, with considerable housing inequalities related to different cohorts, classes and locations [4: 23-24, 12-15].

The postmodern nature of Dutch society partly results from the lengthy process of educational expansion since the Second World War. This has greatly increased the proportion of highly educated people. Almost a third of the population over the age of 25 has now completed tertiary education; and among the youngest cohorts, women outperform men in average educational attainment [16-19]. In addition, the Netherlands has become highly secularised: between 1970 and 2012, the share of people not adhering to any form of organised religion rose from 39% to 70%. Religious participation is more common among older people and migrants [20]. Detraditionalisation can also be seen in the considerable support for progressive ethics (e.g. acceptance of homosexuality, abortion, euthanasia), which fits into a more general post-industrial institutional pattern [21].

Demographically, the number of non-traditional families (single people, lone-parents and co-residents) has increased over time, and from 2011 the large baby boomer cohorts started to reach the statutory retirement age. In 2015 there were 0.7 transfer income recipients (mostly pensioners) for every year of work [22]. According to Statistics Netherlands' definition, in the same year 12% of the Dutch were of non-Western origin, while another 10% had roots in foreign Western countries. This reflects migration waves of low-skilled industrial workers – especially from Turkey and Morocco – and their families; of people from former Dutch colonies (Surinam, Netherlands Antilles) and new EU member states; and of a post-industrial group consisting of asylum-seekers, knowledge workers and foreign students. In recent years an 'ethnic middle class' has emerged among younger second-generation non-Western migrants, who are achieving better labour market positions than their parents [23-26].

In terms of economic regulation and social protection the Netherlands is often regarded as a hybrid regime. On the one hand its formal institutions reflect the Nordic or social-democratic ideal type. For example, all inhabitants with legal status are entitled to social assistance, health insurance and the statutory flat-rate old-age pension. In other respects the Dutch welfare regime resembles the continental or corporatist-conservative ideal type. Occupational pensions supplement the state pension and covered more than three quarters of all pensioners in 2017. However, the amount of the occupational pension depends on the number of years of employment and the sector in which one used to work. Unemployment and disability benefit rights are also conditional on people's employment

history; and active labour market policies, parental leave and childcare facilities are less extensive in the Netherlands than in the Nordic countries. In addition, Dutch labour market regulation fosters a distinction between insiders and outsiders. Employment protection and investment in skills are comparatively high for those on permanent contracts, but lag significantly behind for non-standard workers [27-35]. As in many other countries, a long period of austerity and marketisation policies has reduced the generosity and universality of the Dutch welfare state, especially with regard to social insurance and provisions for the population of working age [36, 37]. Nonetheless, compared to Anglo-Saxon systems, the institutional regime is still quite comprehensive, with pensions and health care in particular performing well in international rankings. The Netherlands hold sixth place on the Global Social Mobility Index, which covers a wide range of collective provisions. This is well above Australia (16th), the UK (24th) and the USA (27th), and just below the Scandinavian countries [38-40]. During the 1990s, economic and labour market performance in the Netherlands was outstanding. The success was often attributed to consultative policy-making and consensus building between the national government, trade unions and national employers' organisations (the 'polder model'). Although this so-called Dutch miracle has now lost much of its lustre, the social partners continue to play an important role in the development and reform of social policy [41, 42]. This is achieved through the Social and Economic Council's (SER) recommendations on government policy; and through collective bargaining agreements on employment terms and pensions, which are declared legally binding for all employees in the economic sector concerned. The Netherlands thus fits in with the 'coordinated market economy' variety of capitalism [43, 44]. In politics, the composition of the Dutch Lower House (Tweede Kamer) is based on party-list proportional representation with no electoral threshold. As a result, all kinds of groups have delegations in this main body of the national legislature, including parties with a populist message or promoting the rights of the elderly, ethnic minorities, farmers and animals. Between the 2012 and 2021 elections, the number of political parties in the Lower House increased from 11 to 17. This political fragmentation tended to result in centre-right or centre-left coalition governments, often consisting of varying alliances of four parties (Conservative Liberals, Christian-Democrats, the Labour Party, Social Liberals). Combined with the influential position of the social partners, the organisation and division of political power evokes complicated bargaining processes that encourage compromises and path dependency in policy-making.

References S1

1. Statistics Netherlands. National accounts 1969-2016: Labour volume by sector; retrieved from opendata.cbs.nl/statline on 12 January 2021.
2. Visser J. The first part-time economy in the world: A model to be followed? J Eur Soc Policy. 2002;12: 23-42.
3. Josten E, Vlasblom JD, Vrooman C. Bevrijd of beklemd? Werk, inhuur, inkomen en welbevinden van zzp'ers. Den Haag: Sociaal en Cultureel Planbureau; 2014.
4. OECD. In it together: Why less inequality benefits all. Paris: Organisation for Economic Co-operation and Development; 2015.
5. Eurofound. New forms of employment. Luxembourg: Publications Office of the European Union; 2015.
6. de Beer P, Conen W. Een halve eeuw arbeidsmarkt. Amsterdam: University of Amsterdam; 2018.
7. Dreher A. Does globalization affect growth? Evidence from a new index of globalization. Appl Econ. 2006;38: 1091-1110.
8. Gygli S, Haelg F, Potrafke N, Sturm J-E. The KOF globalisation index – revisited. Rev Int Organ. 2019;14: 543-574.
9. UNDP. Human development report 2019. New York: United Nations Development Programme; 2019.

10. OECD. Digital economy outlook 2020. Paris: Organisation for Economic Co-operation and Development; 2020.
11. World Bank. World development indicators; retrieved from databank.worldbank.org/source/world-development-indicators on 9 March 2021.
12. van Bavel B, Frankema E. Wealth inequality in the Netherlands, c. 1950-2015: The paradox of a Northern European welfare state. TSEG. 2017;14: 29-62.
13. Vrooman JC, Goderis B, Hoff S, van Hulst B. Measuring poverty in the Netherlands: The generalised reference budget approach. In: Deeming C, editor. Minimum income standards and reference budgets: International and comparative policy perspectives. Bristol: Policy Press; 2020. pp. 169-183.
14. Hochstenbach C, Arundel R. Spatial housing market polarisation: National and urban dynamics of diverging house values. Trans Inst Br Geogr. 2020;45: 464-482.
15. Statistics Netherlands. House price increase accelerating again; retrieved from www.cbs.nl at 11 October 2021.
16. van der Ploeg S. Educational expansion and returns on credentials. Eur Sociol Rev. 1994;10: 63-78.
17. van Hek M, Kraaykamp G, Wolbers MHJ. Family resources and male-female educational attainment: Sex specific trends for Dutch cohorts (1930–1984). Res Soc Stratif Mobil. 2015;40: 29-38.
18. Allen J, Belfi B. Educational expansion in the Netherlands: Better chances for all? Oxf Rev Educ. 2020;46: 44-62.
19. Statistics Netherlands. Population: Highest level of educational attainment and type of education; retrieved from opendata.cbs.nl/statline on 28 April 2021.
20. de Hart J. Geloven binnen en buiten verband: Godsdienstige ontwikkelingen in Nederland. Den Haag: Sociaal en Cultureel Planbureau; 2014.
21. Elchardus M, de Keere K. Institutionalizing the new self; a comparative analysis. Eur Soc. 2010;12: 743-764.
22. CPB. Central economic plan 2021 (annex 14). The Hague: CPB Netherlands Bureau for Economic Policy Analysis, 2021.
23. Statistics Netherlands. Migration background; retrieved from www.cbs.nl/en-gb/onze-diensten/methods/definitions/migration-background on 9 August 2021.
24. CBS. Jaarrapport integratie 2020. Den Haag: Centraal Bureau voor de Statistiek; 2020.
25. Huijnk W. Werk in ontwikkeling? Den Haag: Sociaal en Cultureel Planbureau; 2020.
26. WRR. Samenleven in verscheidenheid: Beleid voor de migratiesamenleving. Den Haag: Wetenschappelijke Raad voor het Regeringsbeleid; 2020.
27. Esping-Andersen G. The three worlds of welfare capitalism. Cambridge: Polity Press; 1990.
28. Esping-Andersen G. Social foundations of postindustrial economics. Oxford: Oxford University Press; 1999.
29. Arts WA, Gelissen JPTM. Models of the welfare state. In: Castles FG, Leibfried S, Lewis J, Obinger H, Pierson C, editors. The Oxford handbook of the welfare state. Oxford: Oxford University Press; 2010. pp. 569-583.
30. Ferragina E, Seeleib-Kaiser M. Welfare regime debate: Past, present, futures? Policy Polit. 2011;39: 583-611.
31. OECD. Protecting jobs, enhancing flexibility: A new look at employment protection legislation. Employment outlook 2013 (chapter 2). Paris: Organisation for Economic Co-operation and Development; 2013. pp. 65-126.
32. Thelen K. Varieties of liberalization and the new politics of social solidarity. New York: Cambridge University Press; 2014.
33. van Kersbergen K, Vis B. Comparative welfare state politics: Development, opportunities, and reform. New York: Cambridge University Press; 2014.
34. Powell M, Yörük E, Bargu A. Thirty years of the three worlds of welfare capitalism: A review of reviews. Soc Policy Admin. 2020;54: 60-87.
35. OECD. Recent trends in employment protection legislation. Employment outlook 2020: Worker security and the COVID-19 crisis (chapter 3). Paris: Organisation for Economic Co-operation and Development; 2020. pp. 168-220.

36. Yerkes M, van der Veen R. Crisis and welfare state change in the Netherlands. Soc Policy Admin. 2011;45: 430-444.
37. Vrooman JC. De maatschappelijke consequenties van het institutionele pad: Regelingen voor werkloosheid, arbeidsongeschiktheid en bijstand, 1980-2015. In: Hirsch Ballin EHM, Jaspers APCM, Knottnerus JA, Vinke H, editors. De toekomst van de sociale zekerheid: De menselijke maat in een solidaire samenleving. Den Haag: Boom Juridisch; 2021. pp. 323-347.
38. Mercer. The Melbourne Mercer Global Pension Index 2016. Melbourne: Mercer/Monash University/Melbourne. Available from: www.mercer.com/our-thinking/mercer-melbourne-global-pension-index.html.
39. Barber RM, Fullman N, Sorensen RJD, Bollyky T, McKee M, Nolte E, et al. Healthcare access and quality index based on mortality from causes amenable to personal health care in 195 countries and territories, 1990-2015: A novel analysis from the global burden of disease study 2015. Lancet. 2017;390: 231-266.
40. World Economic Forum. The global social mobility report 2020: Equality, opportunity and a new economic imperative. Cologny: The World Economic Forum; 2020.
41. Visser J, Hemerijck A. 'A Dutch miracle': Job growth, welfare reform and corporatism in the Netherlands. Amsterdam: Amsterdam University Press; 1997.
42. Keune M, editor. Nog steeds een mirakel? De legitimiteit van het poldermodel in de eenentwintigste eeuw. Amsterdam: Amsterdam University Press; 2016.
43. Hall PA, Soskice D, editors. Varieties of capitalism: The institutional foundations of comparative advantage. Oxford: Oxford University Press; 2001.
44. Wood GT, Allen MMC. Comparing capitalisms: Debates, controversies and future directions. Sociology. 2020;54: 482-500.
