# Supplementary material for: A contemporary class structure: Capital disparities in The Netherlands
Source: PLoS One. 2024 Jan 31;19(1):e0296443. doi: 10.1371/journal.pone.0296443 (PMC10830037; doi:10.1371/journal.pone.0296443)
Supplement: S2 Text — (PDF) [file pone.0296443.s003.pdf]

S2 Text. List of survey items

Translated items of the questionnaire ‘Verschil in Nederland’.

Economic capital
1. Educational attainment: ‘What is the highest level of education you have successfully completed? We speak of successfully completed when a diploma or certificate has been issued indicating that one passed a test for the training; completely attended the training’.
Response categories contain a detailed list of 17 Dutch types of education, running from elementary school (unfinished) to PhD, plus ‘other’ and ‘don’t know’.

2. Disposable household income: n.a. (measured through administrative data)

3. Current labour market position: ‘What is your employment situation? If you have more than one profession or position, choose the job you spend then most time on. If you spend the same amount of time on two or more jobs, choose the job in which you have worked the longest’.
Response categories: never worked; in full-time education or training; retired or pre-retirement, early retirement; no paid job now, but looking for a job; no paid job now, not looking; works as an employee: government or semi-government (e.g. education, health care); works as an employee: commercial company; work as an employee: non-profit-sector; works as self-employed or freelancer; works as assisting spouse or family member

4. Liquid assets: n.a. (measured through administrative data)

5. Home equity: n.a. (measured through administrative data)

Cultural capital
1a. Life style: ‘Where did your farthest holiday trip take you in 2013? A holiday is a stay outside your own home for at least three consecutive nights’.
Response categories: I didn't go on holiday in 2013; the Netherlands; abroad, namely ….

1b. Life style: ‘Did you eat out in a restaurant in 2013 where you have spent more than 100 euros a head (drinks excluded)?’.
Response categories: no; yes, 1 time; yes, 2-3 times; yes, 3-4 times; yes, 5-9 times; yes, more than 10 times; don’t know.

1c. Life style: ‘How often have you been to classical concerts, theater or (art) museums in the past 12 months?’.
Response categories: once a week or more often; once every two weeks; once a month; a few times a year; rarely or never; don't know.

2. Basic digital skills: ‘Are you able to: (i) Work on a computer with a word processor (e.g. Word); (ii) Install a program on a computer; (iii) Arrange the security on a computer (e.g. with a virus scanner or firewall)’.
Response categories: Yes; No

3. Mastery of English: ‘Do you have a good command of the English language?’.
Response categories: (i) no; (ii) yes, a few words (e.g. order something in a pub using a travel guide); (iii) yes, enough to talk about simple and everyday matters (e.g. asking directions); (iv) yes, enough to

have a conversation about everyday topics without preparation (e.g. about family, hobbies); (v) yes, enough to understand the gist of a complex text, and have a spontaneous conversation with a native English speaker; (vi) yes, enough to fully understand complex and long texts, and to express myself fluently in a conversation with a native English speaker; (vii) yes, enough to participate in any conversation or discussion without preparation and deliver a complicated speech; (viii) yes, English is my native language. Condensed version: (1=1) (2,3=2) (4,5=3) (6,7=4) (8=5).

Social capital
1. Strong ties: 'How often do you have contact (i.e. meetings, telephone and written contacts, email, etc.) with: (i) Family relatives (who do not live with you in the same house); (ii) Friends or really good acquaintances; (iii) Neighbors and local residents'.
Response categories: once a week or more often; once every 2 weeks; once a month; less than once a month; rarely or never; does not apply; don't know.

2. Size of the core discussion network: 'With how many people (who do not live with you in the same house) did you discuss important personal matters in the last six months? This does not concern people who provide you with professional help, such as a general practitioner'.
Response categories: with nobody; with 1 person; with 2 or 3 people; with 4 or 5 people; with more than 5 people; does not apply; don't know.

3. Access to people in resourceful positions: 'Do you currently have friends, acquaintances or relatives in one of the following professions? What matters is if you know them personally, outside of their work. (i) A mayor or member of the Second Chamber; (ii) A doctor or lawyer (iii) A director of a commercial company employing more than 10 people; (iv) A high-ranking civil servant, e.g. a municipal secretary or a director at a Ministry; (v) A professional musician, artist or writer'.
Response categories: yes; no.

Person capital
1. Physical capital:
(i) 'Are you currently limited by your health when climbing stairs?'. Response categories: yes, severely limited; yes, a bit limited; no, not limited at all
(ii) 'In general, would you say your health is: excellent; very good; good; fair; poor'.

2. Mental capital:
(i) 'Overall, I have a lot of self-confidence';
(ii) 'Overall, I have pretty negative feelings about myself';
(iii) 'In the past 12 months, have you had periods of at least 14 consecutive days in which you experienced black moods or depression?'.
Response categories (i, ii): scale running from 1 = strongly disagree to 10 = strongly agree + 'don't know'; (iii) yes; no.

3. Aesthetic capital:
(i) 'I think my appearance is just fine as it is';
(ii) 'Most other people think I look good'.
Response categories: (i): scale running from 1 = strongly disagree to 10 = strongly agree + 'don't know'; (ii): scale running from 1 = nobody thinks so to 10 = everybody thinks so + 'don't know'.

4. Body Mass Index:
(i) 'What is your height?' …. cm
(ii) 'What is your weight?' …. kg

Socio-political views
1. Subjective social location: 'In our society there are groups which tend to be towards the bottom layer of our society and groups which tend to be towards the top layer. Below is a scale that runs from the bottom layer (score 0) to the top layer (score 10). Where would you put yourself on this scale at present?'. An illustration of the social ladder (with equal width and rung spacing) was included.

2. Social identification: 'In society various groups are present. Could you indicate to what extent you feel that you are part of the following groups? The influential people; The people with a lot of money; The religious people; The high-educated people; The young people; The Dutch; The attractive people' Each group was scored on a scale running from 1 = not at all to 10 = entirely.

Exploratory factor analysis suggested two subdimensions, resulting in separate scales for identification with rich, influential and high-educated people (α=0.82) and identification with young, native Dutch and attractive people (α=0.68). The item on religious people did not fit in with either of these scales and was discarded.

3/4. Social friction: 'In all countries friction sometimes occurs between social groups. How much friction do you think occurs in the Netherlands between the following groups? Rich and poor people; Employers and employees; Old and young people; Dutch people and ethnic migrants; Various religious groups; High-educated and low-educated people; Sick and healthy people; People who control this country and the rest of the population'. Each pair was scored on a scale running from 1 = no friction at all to 10 = very much friction. The responses on these items form a reliable one-dimensional social friction scale (α=0.88).

5. Societal pessimism: 'Do you think things in the Netherlands are generally going in the right or wrong direction?'
Response categories: 1 = definitely the wrong direction; 2 = slightly more wrong than right direction; 3 = slightly more right than wrong direction; 4 = definitely the right direction.

6. (Dis)contentment on social issues
Discontentment on social issues was calculated as the sum of five subscales (cf. Vrooman 2016: 36-38) on the perceived deficiency of social protection (4 items, α=0.75); aversion to cultural differences (3 items, α=0.80); feelings of political abandonment (3 items, α=0.72); the perceived failure of the Dutch power elite (5 items, α=0.74); and rejection of further EU integration (1 item).
The survey items underlying the five subscales are listed below.

• Perceived deficiency of social protection:
- 'The solidarity between young and old is under pressure in the Netherlands';
Response categories: 1 = strongly disagree; 10 = strongly agree;
- 'Some people think that the government should be more responsible to ensure that everyone gets what he needs (at #1). Others think that people should take more responsibility for taking care of themselves (at #10). Where would you place yourself?'
Response categories: 1 = the government should take more responsibility for ensuring that everyone receives what they need; 10 = people should take more responsibility for taking care of themselves;

- ‘Some people think that the differences in incomes in our country should be reduced (at #1). Others that they should get larger (at #10). Where would you place yourself?’
Response categories: 1 = differences in income should become smaller; 10 = differences in income should become larger;
- ‘Some people think that the differences in wealth in our country should be reduced (at #1). Others that they should get larger (at #10). Where would you place yourself?’
Response categories: 1 = differences in wealth should become smaller; 10 = differences in wealth should become larger.

• Aversion to cultural differences:
- ‘Differences in norms and values between natives and migrants are a problem’.
Response categories: 1 = strongly disagree; 10 = strongly agree;
- ‘Differences in religion between natives and migrants are a problem’.
Response categories: 1 = strongly disagree; 10 = strongly agree;
- ‘Some people think that migrants should be able to live in the Netherlands while retaining their own culture (at #1). Others think that they should fully adapt to Dutch culture (at #10). Where would you place yourself?’.
Response categories: 1 = retain own culture; 10 = adapt fully.

• Feelings of political abandonment:
- ‘The government doesn't do enough for people like me’
Response categories: 1 = strongly disagree; 5 = strongly agree
- ‘People like me have no influence at all over what the government is doing’
Response categories: 1 = strongly disagree; 5 = strongly agree
- ‘MPs and ministers don't care very much what people like me think’
Response categories: 1 = strongly disagree; 5 = strongly agree

• Perceived failure of the Dutch power elite:
- ‘How much trust do you currently have in the Second Chamber?’
Response categories: 1 = no trust at all; 10 = complete trust
- ‘The elite don't understand a thing about me’
Response categories: 1 = strongly disagree; 10 = strongly agree.
- ‘The elite mostly look after their own interests’
Response categories: 1 = strongly disagree; 10 = strongly agree
- ‘People who belong to the elite have usually achieved their special position in an honest way’
Response categories: 1 = strongly disagree; 10 = strongly agree
- ‘Do you think the elite contribute to the fact that the Netherlands is generally heading in the right or wrong direction?
Response categories: 1 = Because of the elite, the Netherlands is generally heading in the wrong direction; 10 = Because of the elite, the Netherlands is generally heading in the right direction

• Rejection of further European integration:
- ‘Some people think that European integration should go even further (at # 1). Others think that European integration has already gone too far (at #10). Where would you place yourself?’
Response categories: 1 = European integration must go further; 10 = European integration has already gone too far (converted into standard scores).

7. Trust in other people: 'Most people can be trusted'.
Response categories: 1 = strongly disagree; 10 = strongly agree.

Personal values
'Below we briefly describe some people. Please read each description and indicate how much each person is or is not like you. In each of the following statements, 'she' refers to an imaginary person'.
- 'It is important to her to listen to people who are different from her. Even when she disagrees with them, she still wants to understand them.' [Universalism]
- 'It is important to her to help the people around her. She wants to care for their well-being.' [Benevolence]
- 'It is important to her always to behave properly. She wants to avoid doing anything people would say is wrong.' [Conformity]
- 'Tradition is important to her. She tries to follow the customs handed down by her religion or her family.' [Tradition]
- 'It is important to her that people do what she says. She wants to get respect from others.' [Power I]
- 'It is important to her to be rich. She wants to have a lot of money and expensive things.' [Power II]
- 'Being very successful is important to her. She hopes people will recognise her achievements.' [Achievement]
- 'Having a good time is important to her. She likes to "spoil" herself.' [Hedonism]

Response categories: 1 = not like me at all; 2 = not like me; 3 = not very much like me; 4 = somewhat like me; 5 = like me; 6 = very much like me.

Male respondents were given a version of the questionnaire tailored to their gender.

These 8 items are a subset of the original 21-item Portrait Values Questionnaire. Universalism and benevolence combine in Schwartz's theory into the higher-order value of 'self-transcendence'. Tradition and conformity cover two of the three aspects of 'conservation'. When combined, according to Schwartz power and achievement theoretically indicate 'self-enhancement', while hedonism is one element of 'openness to change'. We found an adequate scale for self-enhancement plus hedonism (4 items, α=0.73), and weaker ones for conservation (2 items, α=0.58) and self-transcendence (2 items, α=0.53).

Well-being
1. Satisfaction with life: 'On a scale from 1 to 10 can you indicate how satisfied you are with the life you lead at the moment? A score of 1 refers to completely dissatisfied and a 10 to completely satisfied.'
2. Making ends meet: 'With your total net household income, how well can you to make ends meet nowadays, i.e. pay for your usual necessary expenses?' 1 = very difficult; 6 = very easy.

All survey questions on socio-political views (except subjective social location), personal values and well-being included a 'don't know' option. These cases have been treated as missing.
