# Supplementary material for: A contemporary class structure: Capital disparities in The Netherlands
Source: PLoS One. 2024 Jan 31;19(1):e0296443. doi: 10.1371/journal.pone.0296443 (PMC10830037; doi:10.1371/journal.pone.0296443)
Supplement: S3 Text — (PDF) [file pone.0296443.s004.pdf]

S5 Text. Residential separation of capital groups

Figure S5A reveals some residential separation of the six capital groups at the scale of labour market regions and the three largest Dutch municipalities. The established upper echelon and privileged younger people are overrepresented in the 'northern branch' of the Randstad conurbation (i.e. the Western part of the Netherlands, including the major cities of Amsterdam, Rotterdam, The Hague and Utrecht). The Amersfoort region (#1) and the city of Amsterdam (#2) have the most favourable population composition. Outside the Randstad, total capital is also high in the region around Nijmegen (#3) and in the so-called Brainport of Eindhoven (#11). This 'Dutch Silicon Valley' combines a high share of privileged younger people with an overrepresentation of insecure workers.

Figure S5A. Residential separation of capital groups in 35 labour market regions and the three largest municipalities

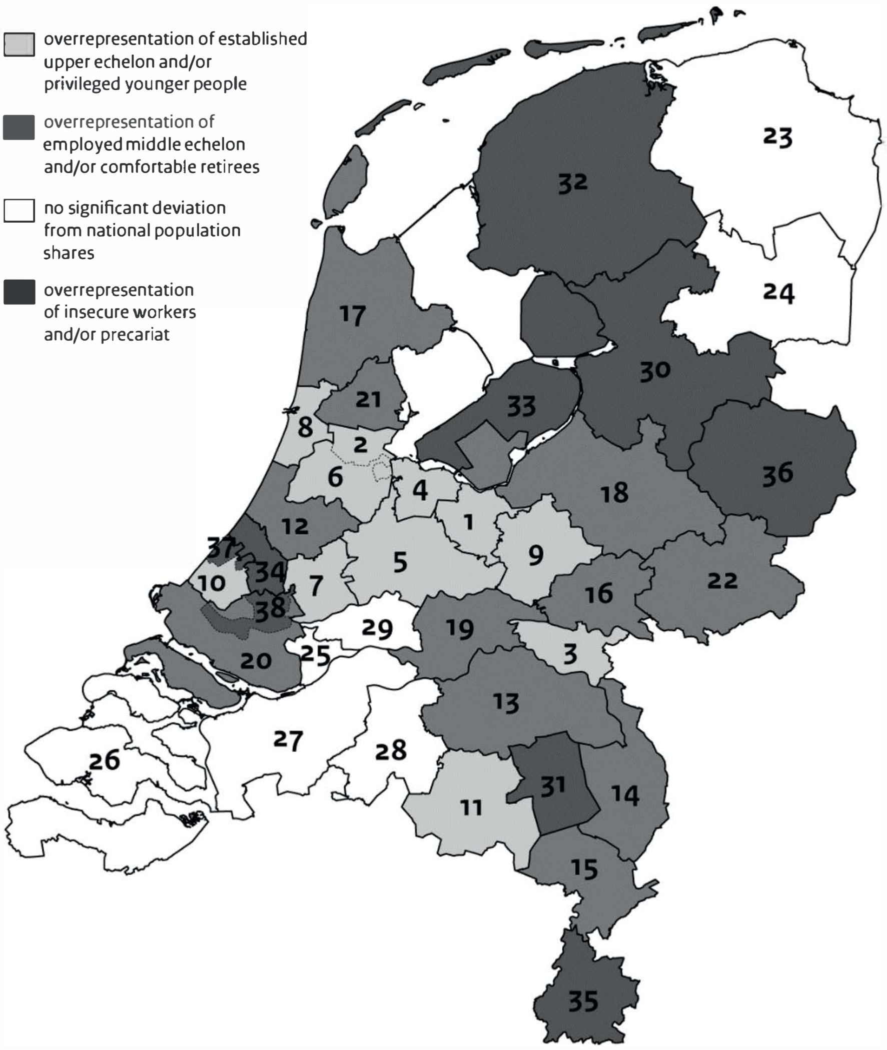

#: ranking, 1 = area with most favourable composition by capital group, 38 = area with least favourable composition.
Republished from *De ruimtelijke dimensie van Verschil in Nederland* under a CC BY license, with permission from Sociaal en Cultureel Planbureau, original copyright 2017.

The less urbanised areas surrounding the Randstad (plus the Holland-Rijnland labour market region within it) are home to a relatively large number of people in the employed middle echelon or comfortable retirees group (#12-22). The labour market regions in the southwest and northeast of the Netherlands do not deviate markedly from the general pattern (#23-29). The areas with the most unfavourable composition - due to an overrepresentation of insecure workers and/or the precariat - are located in Friesland, Flevoland, the eastern Netherlands, South Limburg and the 'southern branch' of

the Randstad (#30-38). Among the three largest municipalities, the contrast between the favourable population composition of Amsterdam and the unfavourable one of The Hague (#37) and Rotterdam (#38) is striking.

At a smaller scale, the precariat and insecure workers are overrepresented in postcode areas with low social status and in the 40 designated ‘problem neighbourhoods’ identified in a national policy programme (Figure S5B; data obtained from ViN, Statistics Netherlands and the Bisnode database). Privileged younger people also make up a relatively high proportion of the population here, which is logical given their limited income and the lower rents in these places. The established upper echelon is overrepresented in postcode areas with high social status and in neighbourhoods with the highest fiscal real estate value. The employed middle echelon and comfortable retirees are almost as rare in unfavourable neighbourhoods as the established upper echelon, but less represented in better postcode areas.

Figure S5B. Residential separation of capital groups in different types of postcode areas*

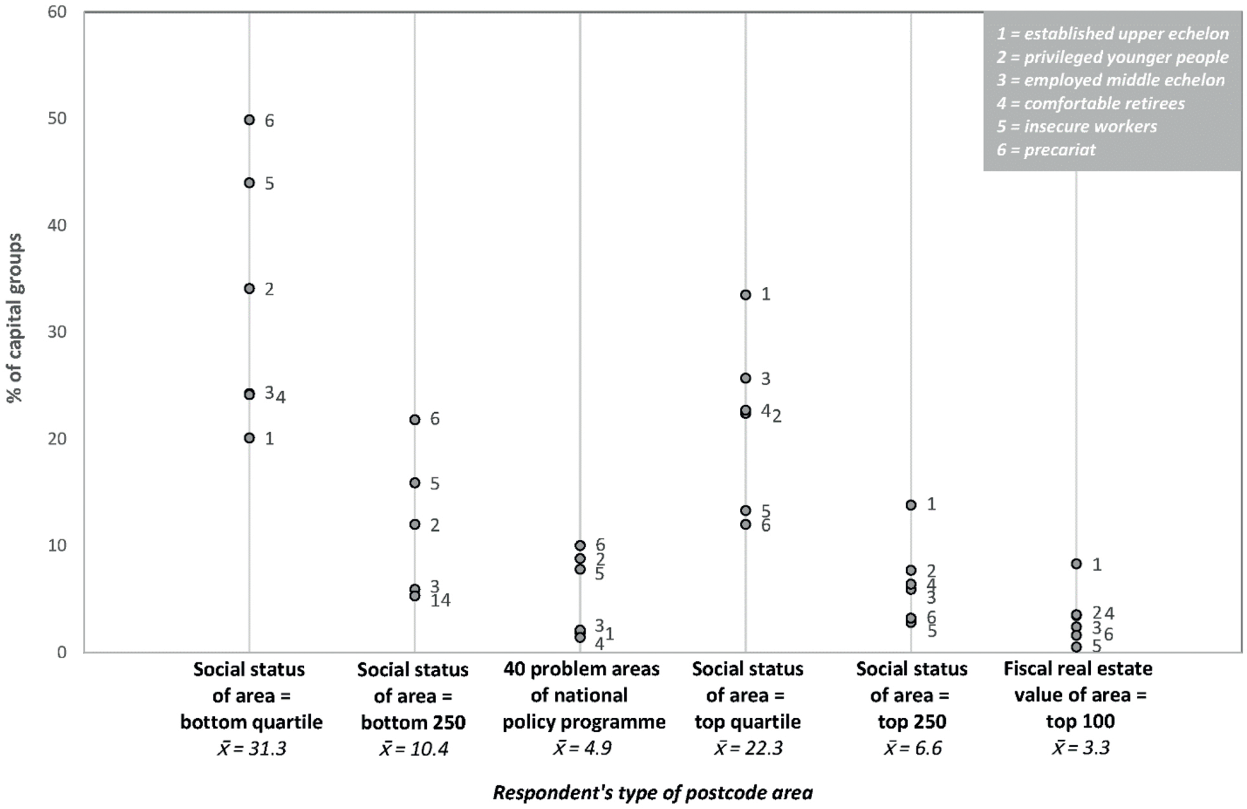

* Social status variables: quartiles and bottom/top 250 are based on the ranking of 3584 four-digit postcode areas

While a pattern is discernable, the separation of the capital groups by postcode area is by no means perfect. A large majority of the established upper echelon (91%) do not live in the 100 neighbourhoods with the highest average fiscal real estate values; only 13% of the precariat are in a recognised problem area, and almost half do not live in the 250 neighbourhoods with the lowest social status.

Overall, there appears to be evidence of sorting in labour market regions and type of postcode area, but not of a complete residential segregation of the capital groups. Follow-up research with more observations could provide a more detailed picture.
