# Supplementary material for: A contemporary class structure: Capital disparities in The Netherlands
Source: PLoS One. 2024 Jan 31;19(1):e0296443. doi: 10.1371/journal.pone.0296443 (PMC10830037; doi:10.1371/journal.pone.0296443)
Supplement: S4 Text — (PDF) [file pone.0296443.s005.pdf]

S6 Text. Educational homogamy and absence of intergenerational mobility

Educational homogamy

The correlation between the level of educational attainment of spouses is fairly strong (r=0.57), with more than half of the couples having the same type of grade. Educational homogamy is widespread in the established upper echelon (68%). This mainly concerns the relationships of high educated people, and this is also the largest homogeneous group among the privileged younger people. In the precariat, marriages or partnerships between low-educated people are common: in a quarter of all couples, both partners have completed no more than primary education. In the other five capital groups, homogamy at the lowest level of education is virtually absent.

Table S6. Educational homogamy and absence of educational and occupational intergenerational mobility, by respondent's latent class

	total sample	Established Upper Echelon	Privileged Younger People	Employed Middle Echelon	Comfortable Retirees	Insecure Workers	Precariat
Educational homogamy[a]							
Homogamous (total)	57%	68%	53%	55%	55%	50%	59%
- both spouses primary education or less	3%	0%	0%	0%	1%	0%	24%
- both lower secondary or both upper secondary	31%	9%	6%	37%	48%	46%	35%
- both spouses tertiary education	23%	59%	47%	17%	6%	5%	0%
correlation (*r* education respondent x spouse)	0.57						
uniform association parameter	0.58						
Absence of educational mobility[a]							
No mobility (total)	39%	36%	44%	29%	45%	38%	57%
- both respondent and father primary education or less	4%	0%	0%	0%	3%	3%	33%
- both lower secondary or both upper secondary	20%	5%	7%	19%	38%	29%	23%
- both respondent and father tertiary education	15%	31%	37%	10%	4%	5%	1%
correlation (*r* education respondent x father)	0.43						
uniform association parameter	0.42						
Absence of occupational mobility[b]							
No data (% of all respondents)[c]	16%	5%	10%	9%	12%	28%	42%
No mobility (total)	42%	50%	42%	40%	36%	37%	54%
- both respondent and father lower occupation	6%	0%	1%	4%	6%	9%	29%
- both respondent and father middle occupation	17%	10%	6%	19%	22%	21%	23%
- both respondent and father higher occupation	20%	40%	36%	17%	8%	7%	3%
correlation (*r* occupation respondent x father)	0.27						
uniform association parameter	0.33						

[a] 4 categories: primary education or less; lower secondary; higher secondary; tertiary.
[b] 3 categories: lower occupations (ISCO-08 8-9); middle occupations (ISCO-08 4-7); higher occupations(ISCO-08 1-3). Father's occupation when respondent was 15 years of age; for non-working respondents: level of previous occupation.
[c] Father deceased or non-working when respondent was 15 years of age; father's occupation unknown to respondent; respondent has never worked.

Absence of educational mobility

The statistical association between the educational level of the respondents and that of their father is less pronounced (r=0.43). Despite the long-term trend towards educational expansion, a large share of respondents (39%) have not experienced intergenerational educational mobility. Among the precariat, this even applies to a majority (57%), with both generations frequently having completed no more than primary education (33%). Educational immobility is also relatively high among the comfortable retirees and privileged younger people, although here both generations have usually attained the secondary or tertiary level. It is least common among the employed middle echelon, where downward educational mobility is above average. Immobility is relatively low in the established upper echelon; as with the privileged younger people, this mostly concerns highly educated fathers and children.

Absence of occupational mobility

The occupational level of the respondent was measured using the International Standard Classification of Occupations (v2008 version; open questions converted to a four-digit code). A single closed question was used for parental occupation (‘What kind of work did your father perform when you were 15 years old?’) [1]. As the father’s occupational level was assessed in less detail than that of the respondent, we use a broad classification of low, intermediate and higher jobs.

It turned out to be impossible to assess occupational mobility for 16% of respondents, and this particularly occurred among the insecure workers and the precariat (28-42%). The high drop-out rate is partly due to the fact that the father was unemployed or dead when the respondent was 15 years old. However, it is mainly because the respondent did not know what their father’s job was at that time. That may be the result of memory issues, but it is also possible that the father was unknown or absent. For the remaining valid observations, the correlation between the occupational levels of the respondent and that of their father is 0.27, which is weaker than for homogamy and mobility in educational attainment. On average, the occupational level of father and child is the same in 42% of cases. In the precariat and the established upper echelon this applies to a majority of cases. In 29% of cases in the precariat, both generations had a low occupation, while in the established upper echelon 40% had a higher occupation in both generations. The latter was also common among privileged younger people.

Relative homogamy and mobility

The uniform association parameters in table S6 take into account the divergent supply in each educational or occupational class, as shown in the marginal distributions [cf. 2]. This corrects for cohort effects, such as the comparatively large share of highly educated women among privileged younger people. Relative educational homogamy and the two forms of relative occupational mobility prove to be statistically significant.

Conclusion

In terms of stability, the precariat and the established upper echelon especially stand out. The group with the least capital combines a high degree of homogamy with limited intergenerational educational and occupational mobility. Like their partners and fathers, members of the precariat mostly end up at the lowest educational and occupational levels. The established upper echelon also exhibits high homogamy and low intergenerational mobility. Here, however, partners are often found among the respondent’s own circle of well-educated people, and high parental educational and occupational levels tend to be reproduced. This pattern also occurs - albeit to a lesser extent - among privileged younger people.

References S6

1. de Vries J, Ganzeboom HBG. Hoe meet ik beroep? Open en gesloten vragen naar beroep toegepast in een statusverwervingsmodel. Mens en Maatschappij. 2008;83: 71-96.
2. Goodman LA. Simple models for the analysis of association in cross-classifications having ordered categories. J Am Stat Assoc. 1979;74: 537-552.
