# Supplementary material for: A contemporary class structure: Capital disparities in The Netherlands
Source: PLoS One. 2024 Jan 31;19(1):e0296443. doi: 10.1371/journal.pone.0296443 (PMC10830037; doi:10.1371/journal.pone.0296443)
Supplement: S5 Text — (PDF) [file pone.0296443.s006.pdf]

S9 Text. Distribution of resources by age

In theory, the age-relatedness of the resources of the six capital groups can indicate cohort, period and ageing effects. Cohort effects in the age distribution of resources occur, for example, when younger groups tend to be better educated than older ones because they have benefited more from the process of educational expansion. Ageing effects may show in higher mean wealth among the elderly, who have generally had more time to accumulate liquid assets through work, profits, returns on investments, inheritances etc. Period effects are also conceivable: the distribution of unemployment by age may be different in times of economic prosperity and than in periods of decline, among other things because school leavers are less likely to be hired during a recession.

Empirically, we will not find any period effects in our data (as there is only one point of measurement), but only the interaction of age and cohort effects. The various capital indicators can be classified according to the shape of their distribution by age group (Fig S9). Many resources show a rather steady downward trend. This is the case for indicators of educational attainment, current labour market position, digital skills and language proficiency. Older groups are less likely than younger ones to have benefited from educational expansion, to be in paid employment, to possess basic digital skills and to have acquired a good mastery of English. A decline in resources by age group is also observed in physical health, the body mass index and the three forms of social capital. This can partly be explained by age effects: as people grow older, they become physically less healthy, while their social networks shrink. Some of the nine resource indicators with a downward trend first rise at a young age, when people complete their education, enter the labour market, and start to build their social networks.

Liquid assets and home equity grow steadily, except for an initial decline in wealth that is largely due to young adults leaving the parental household. The upward trend probably reflects both ageing processes - as they become older, people have had more time to inherit or accrue wealth - and cohort effects: some age groups were able to buy their first home in more favourable market conditions than others. Mental health also generally improves when people are in a higher age group, but this trend is reversed if they are seventy-five or older. Finally, household income and life style scores rise initially, but these resources decline once people reach retirement age. Aesthetic capital shows little variation by age group, with no clear trend.

The combined resource patterns by age group result in a moderately downward trend in total capital. On a scale of zero to one, the 18-34-year-olds achieve an average total score of 0.59. This falls to 0.45 for those aged 75 and over (Fig S9).

S9 Figure. Mean scores on capital indicators by type of age distribution

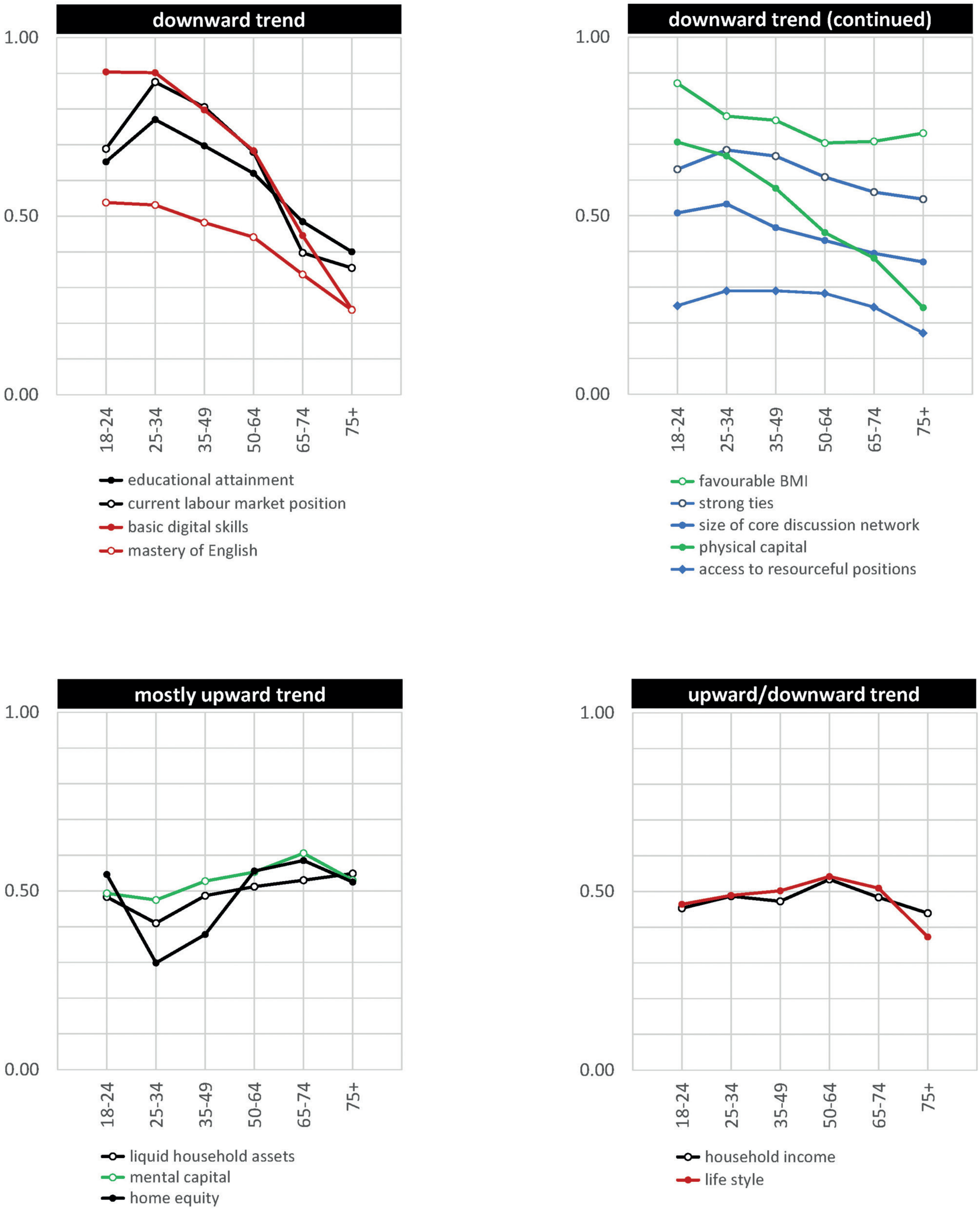

S9 Figure (continued).

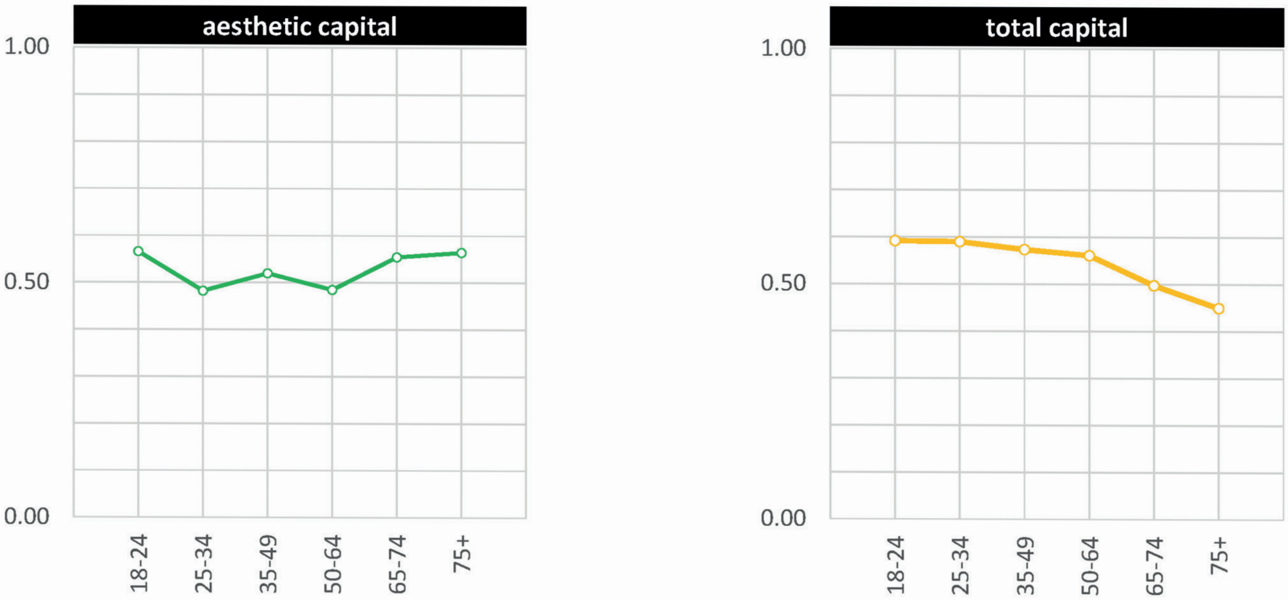

Line colours

black economic capital indicator
red cultural capital indicator
blue social capital indicator
green person capital indicator
orange total capital
