# Supplementary material for: A contemporary class structure: Capital disparities in The Netherlands
Source: PLoS One. 2024 Jan 31;19(1):e0296443. doi: 10.1371/journal.pone.0296443 (PMC10830037; doi:10.1371/journal.pone.0296443)
Supplement: S6 Text — (PDF) [file pone.0296443.s007.pdf]

S10 Text. Capital groups and occupational classes

A bivariate analysis shows that capital groups and occupational classes are related, but by no means identical (FigS10). Not everyone with a higher occupation belongs to the established upper echelon or to the privileged younger people: a considerable share of the higher and lower controllers are in the middle in terms of their resources (43%) or even end up among the insecure workers or the precariat (11%). The self-employed are regarded as a middle class in the EGP scheme; yet nearly one in four belong to the most resourceful capital group, the established upper echelon. In addition, 18% of the self-employed without staff are insecure workers, suggesting a high degree of heterogeneity within this occupational class. When it comes to resources, those working in the lower sales and services sectors are not always in the middle either. In line with the notion of an emerging post-industrial service class, 41% of this group are insecure workers or in the precariat. Of the (un)skilled and agfricultural labourers at the bottom of the occupational structure, a slight majority (51%) belong to the insecure workers or the precariat. The rest of these traditional working classes are mostly in the employed middle echelon or comfortable retirees. Finally, more than two thirds of the group with no work experience consist of people in the precariat and comfortable retirees. This probably reflects the rather large group that ends up on disability insurance and social assistance due to permanent work incapacity, and the low labour participation of women in the older generations.

S10 Figure. Capital groups and occupational classes (EGP; current or last employment); bivariate population shares and nPCA category quantifications on one underlying dimension

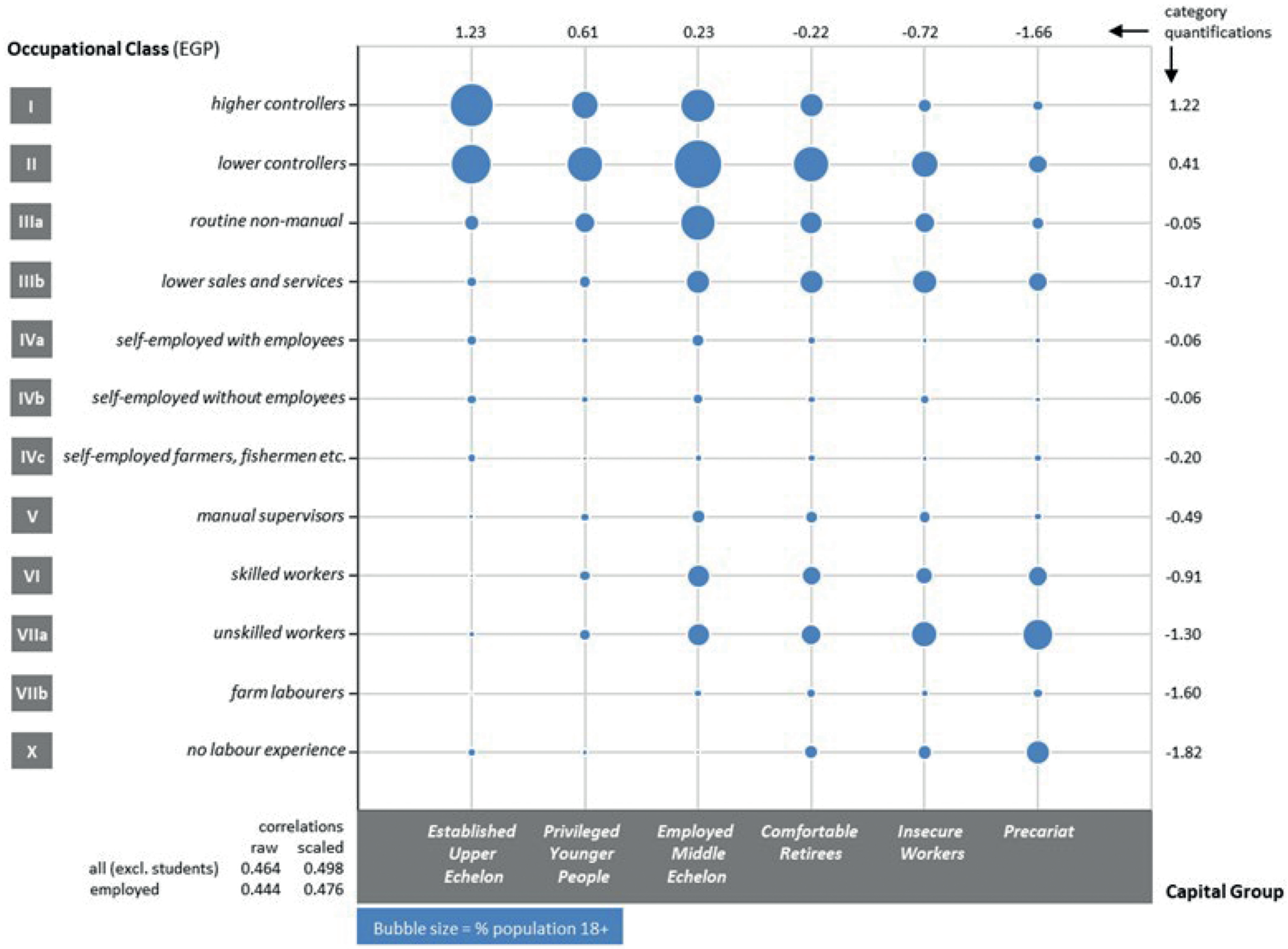

In terms of gender, women are strongly overrepresented in lower sales and services occupations and among those with no work experience. They are much less likely to be higher controllers, self-employed without staff, farmers, manual supervisors and (un)skilled workers. In contrast, there are only two significant gender differences among capital groups: a majority of the precariat are women (59%), while the established upper echelon is predominantly male (57%).
