# Supplementary material for: A contemporary class structure: Capital disparities in The Netherlands
Source: PLoS One. 2024 Jan 31;19(1):e0296443. doi: 10.1371/journal.pone.0296443 (PMC10830037; doi:10.1371/journal.pone.0296443)
Supplement: S7 Text — (PDF) [file pone.0296443.s008.pdf]

S11 Text. Correlations of subjective measures with objective disparities and voting intentions

In an additional analysis, new scales were constructed for the two main clusters of variables that emerged from Fig 3. The capital group variable was not included at this stage. The first measure (Cronbach's alpha=0.74) is based on the indicators for subjective location on the social ladder, social frictions, societal optimism, contentment on social issues, trust in others, life satisfaction, and being able to make ends meet. The second measure (Cronbach's alpha=0.63) consists of the two identification scales (young/attractive/native Dutch; rich/influential/high-educated) and the personal value scale pertaining to self-enhancement and hedonism. Missing values were randomly imputed through nPCA. The two new scales are moderately correlated (r=0.32).

We then calculated the correlations of these scales with the capital group and the objective traits (all in their original coding pattern) and voting intentions discussed in §4.2. Voting intentions were re-classified into four variables. People who opted for a certain political party were positioned on two dimensions: Economic Left/Right and Cultural Conservative/Progressive. Scores for these dimensions were obtained from *Kieskompas 2017*, which ranked Dutch political parties based on the content of their national election programmes (cf. tweedekamer2017.kies- kompas.nl/nl/). Furthermore, we included variables that distinguish between voters vs. nonvoters, and voters with a party preference vs. swing voters.
Respondent scores on the two underlying dimensions of Fig 2 were also included. These indicate the more general 'age-based capital disparities' (age-dependent resources, and their intersection with religiousness, the presence of minor children, etc.) and 'general social hierarchy' (the combination of resource levels, EGP, having a permanent labour contract, ethnic origin, gender, etc.).

S11 Table. Correlations of two subjective measures with objective traits and voting intentions

	Subjective class hierarchy (excl. identification and personal values)		Identification and self-enhancement/ hedonism	
	correlation	CI	correlation	CI
Class and age				
Capital group	**0.49**	[0.46 0.52]	**0.40**	[0.37 0.43]
Occupational class (EGP)	**0.36**	[0.33 0.40]	0.22	[0.19 0.26]
Age group	-0.02	[-0.06 -0.02]	**-0.35**	[-0.39 -0.32]
Age-based capital disparities (=D1 Fig 2)	-0.05	[-0.08 -0.01]	**-0.30**	[-0.34 -0.27]
General social hierarchy (=D2 Fig 2)	**0.50**	[0.47 0.52]	0.29	[0.25 0.32]
Voting intentions				
Non-voters	-0.27	[-0.31 -0.24]	-0.14	[-0.18 -0.10]
Swing voters	-0.12	[-0.16 -0.08]	-0.09	[-0.13 -0.05]
Left/Right party preference	0.18	[0.14 0.22]	0.26	[0.22 0.30]
Cultural-Conservative/Progressive party preference	0.09	[0.04 0.13]	-0.08	[-0.13 -0.04]
Other				
Ethnic origin (high=non-Western)	-0.19	[-0.22 -0.15]	-0.02	[-0.05 0.02]
Labour contract (high=permanent)	0.13	[0.08 0.18]	-0.10	[-0.15 -0.05]
Gender (high=female)	-0.03	[-0.06 -0.01]	-0.09	[-0.13 -0.05]
Household type (high=couple with children)	0.13	[0.10 0.17]	0.07	[0.03 0.11]
Religion (high=religious)	0.01	[-0.02 0.05]	-0.04	[-0.08 -0.01]

On both subjective components, the relationship with the six capital groups is found to be very pronounced. It is worth noticing that the correlations of the two components with the capital typology (r=0.49/0.40) are clearly stronger than those with the EGP classification (r=0.36/0.22). Socio-political views, well-being and personal values are therefore generally more intertwined with the contemporary resource-based class structure than with traditional occupation-based class distinctions. On the first

subjective measure, “general social hierarchy” has almost the same correlation (*r*=0.50) as the capital group typology; on the second, the correlation is much weaker (*r*=0.29). Thus, this more comprehensive measure does not lead to more insight into the views people hold than the resource-based classification.

Age group correlates mainly with the second subjective measure: older people identify less strongly than younger people with the two types of social groups, and are less focused on self-enhancement and hedonism. However, this contingency (*r*=-0.35) does not exceed the correlation with the capital group typology. In addition, the simple breakdown by age group correlates more strongly with the second measure than the generic dimension of "age-based capital disparities and related factors” (*r*=-0.30).

The relationships with the other objective traits are weaker. On the first measure, non-Western migrants, people on temporary labour contracts and smaller households attain somewhat lower scores than their counterparts (*r*=0.13 to 0.19). The correlations with the second measure indicate that females and temporary workers identify slightly less with the young/attractive/native Dutch and the rich/influential/high-educated people, and are also somewhat less inclined towards self-enhancement and hedonism.

The voting intentions on the Left-Right and Cultural-Conservative/Progressive dimensions may say something about whether the two subjective measures reflect more general orientations, ideologies and experiences that people are likely to express in the voting booth. With regard to the left-right distinction, this indeed seems to be the case to some extent. As party preferences becomes more right-wing, respondents place themselves higher on the social ladder, see fewer social frictions, and are more optimistic about how the Netherlands is doing. Voters of more right-wing parties also tend to have more trust in other people, to be more satisfied with their own lives, and to be more likely to think they can make ends meet. By extension, the more right-wing people vote, the stronger they identify with the young/attractive/native Dutch and the rich/influential/high-educated people, and the more they value self-enhancement and hedonism. However, the correlations of the two subjective measures with leftist or right-wing voting intentions are not very pronounced (*r*=0.18/0.26), so it certainly is not a one-to-one relationship. Associations with culturally conservative and progressive voting intentions are even lower (*r*=0.09/-0.08).[1]

[1] On the individual items that underly the two measures, the bivariate correlation of Left/Right party preferences with the scale of people’s (dis)contentment on social issues is not statistically significant (*r*=0.01). However, the link of that scale – consisting of the perceived deficiency of social protection; aversion to cultural differences; feelings of political abandonment; the perceived failure of the Dutch power elite; rejection of further EU integration – with the Cultural-Conservative/Progressive voting intentions is rather substantial (*r*=0.25).
