# Supplementary material for: A contemporary class structure: Capital disparities in The Netherlands
Source: PLoS One. 2024 Jan 31;19(1):e0296443. doi: 10.1371/journal.pone.0296443 (PMC10830037; doi:10.1371/journal.pone.0296443)
Supplement: S1 Table — (PDF) [file pone.0296443.s009.pdf]

S3 Tables. Fit measures of the latent class analysis

S3A Table. Information criteria

# of classes	Bayesian Information Criterion (BIC)	Akaike Information Criterion (AIC)
**1**	110492.393	110186.891
**2**	105786.178	105169.184
**3**	104984.504	104056.017
**4**	104542.613	103302.634
**5**	104343.196	102791.724
**6**	104282.742	102419.778
**7**	104411.943	102237.487

S3B Table. Average latent class probabilities for most likely latent class membership (row) by latent class (column)

**Latent Class**	**1** EUE	**2** PYP	**3** EME	**4** CoR	**5** InW	**6** Pre
**1** Established Upper Echelon	**0.863**	0.035	0.077	0.024	0.002	0.000
**2** Privileged Younger People	0.059	**0.801**	0.085	0.007	0.048	0.000
**3** Employed Middle Echelon	0.054	0.049	**0.812**	0.034	0.050	0.001
**4** Comfortable Retirees	0.028	0.004	0.051	**0.835**	0.027	0.055
**5** Insecure Workers	0.001	0.039	0.082	0.034	**0.813**	0.032
**6** Precariat	0.000	0.000	0.001	0.073	0.030	**0.897**

Entropy-R^2 of the BIC-minimised analysis (6 classes) = 0.763
