# Supplementary material for: A contemporary class structure: Capital disparities in The Netherlands
Source: PLoS One. 2024 Jan 31;19(1):e0296443. doi: 10.1371/journal.pone.0296443 (PMC10830037; doi:10.1371/journal.pone.0296443)
Supplement: S2 Table — (PDF) [file pone.0296443.s010.pdf]

S4 Tables. Capital indicators: mean scores by latent class and correlations

S4A Table. Mean scores of latent classes (minimum=0; maximum=1)

Capital indicators	Established Upper Echelon	Privileged Younger People	Employed Middle Echelon	Comfortable Retirees	Insecure Workers	Precariat
Economic capital						
- educational attainment	0.905	0.863	0.709	0.469	0.548	0.227
- current labour market position	0.768	0.822	0.887	0.466	0.622	0.396
- disposable household income*	0.678	0.411	0.567	0.507	0.322	0.325
- liquid household assets	0.645	0.351	0.546	0.567	0.321	0.421
- home equity	0.638	0.276	0.487	0.664	0.245	0.346
Cultural capital						
- life style	0.843	0.681	0.480	0.513	0.237	0.224
- basic digital skills	0.877	0.971	0.880	0.464	0.837	0.133
- mastery of English language	0.609	0.648	0.489	0.335	0.442	0.147
Social capital						
- strong ties	0.719	0.688	0.675	0.620	0.624	0.396
- core discussion network	0.586	0.647	0.469	0.400	0.379	0.253
- resourceful positions	0.555	0.448	0.180	0.196	0.186	0.076
Person capital						
- physical capital	0.677	0.708	0.658	0.391	0.405	0.199
- mental capital	0.701	0.550	0.540	0.614	0.337	0.344
- aesthetic capital	0.608	0.673	0.470	0.597	0.362	0.411
- favourable body mass index	0.825	0.869	0.790	0.781	0.638	0.585

* Adjusted using the Statistics Netherlands equivalence scale.

S4B Table. Correlations of capital indicators with educational attainment, the four capital types and total capital*

Capital indicators	educational attainment	economic capital	cultural capital	social capital	person capital	total capital
Economic capital						
- educational attainment		**0.62**	**0.59**	**0.36**	0.24	**0.60**
- current labour market position	**0.31**	**0.56**	**0.35**	0.13	0.17	**0.37**
- disposable household income*	**0.28**	**0.61**	**0.30**	0.18	0.16	**0.37**
- liquid household assets	0.13	**0.50**	0.11	0.09	0.12	**0.25**
- home equity	0.04	**0.51**	0.05	0.05	0.09	0.20
Cultural capital						
- life style	**0.33**	**0.34**	**0.72**	**0.32**	0.22	**0.58**
- basic digital skills	**0.47**	**0.34**	**0.76**	0.28	0.19	**0.52**
- mastery of English language	**0.56**	**0.37**	**0.73**	**0.38**	0.23	**0.58**
Social capital						
- strong ties	0.16	0.14	0.25	**0.66**	0.13	**0.35**
- core discussion network	0.27	0.19	**0.31**	**0.73**	0.12	**0.43**
- resourceful positions	**0.35**	0.23	**0.39**	**0.69**	0.16	**0.50**
Person capital						
- physical capital	0.29	0.32	**0.36**	0.19	**0.63**	**0.54**
- mental capital	0.13	0.18	0.17	0.10	**0.65**	**0.42**
- aesthetic capital	0.08	0.05	0.10	0.12	**0.69**	**0.37**
- favourable body mass index	0.18	0.20	0.18	0.10	**0.54**	**0.38**

* Pearson's *r* (pairwise); all correlations are statistically significant (p<.05)
