# Supplementary material for: A contemporary class structure: Capital disparities in The Netherlands
Source: PLoS One. 2024 Jan 31;19(1):e0296443. doi: 10.1371/journal.pone.0296443 (PMC10830037; doi:10.1371/journal.pone.0296443)
Supplement: S3 Table — (PDF) [file pone.0296443.s011.pdf]

S8 Supplementary table Fig 2

S8 Table. Mean scores of capital groups on the transformed variables, by dimension*

	min	max	Capital group					
			Established Upper Echelon	Privileged Younger People	Employed Middle Echelon	Comfortable Retirees	Insecure Workers	Precariat
**Dimension I**								
Age group	-0.90	1.75	-0.05	-0.60	-0.49	0.97	-0.46	0.78
Household type	-0.68	0.73	-0.05	0.01	-0.23	0.32	-0.15	0.22
Labour contract	-0.20	0.05	0.02	-0.03	0.01	0.02	-0.03	-0.03
Voting intention	-0.57	1.87	-0.03	-0.20	-0.08	0.28	-0.06	0.09
Religion	-0.26	0.60	-0.03	-0.11	-0.07	0.11	0.02	0.11
Occupation (EGP)	-0.16	0.28	-0.07	-0.03	-0.01	0.01	0.04	0.10
Ethnic origin	-0.44	0.06	0.04	-0.03	0.03	0.04	-0.06	-0.07
Gender	-0.03	0.03	-0.00	-0.00	-0.00	0.00	0.01	0.00
**Dimension II**								
Age group	-0.00	0.00	0.00	0.00	0.00	-0.00	0.00	-0.00
Household type	-0.97	0.30	0.11	-0.12	0.06	0.07	-0.11	-0.11
Labour contract	-0.58	0.15	0.06	-0.08	0.04	0.06	-0.10	-0.07
Voting intention	-0.91	1.12	0.34	0.01	0.04	0.07	-0.27	-0.28
Religion	-0.16	0.07	0.01	0.03	0.02	-0.03	0.00	-0.03
Occupation (EGP)	-1.94	1.08	0.50	0.24	0.09	-0.09	-0.28	-0.68
Ethnic origin	-1.63	0.23	0.15	-0.11	0.12	0.15	-0.23	-0.25
Gender	-0.32	0.33	0.05	0.01	0.02	-0.01	-0.05	-0.03

* Transformed variables are mean-centered at 0.00
